# Analysis of β-lactone formation by clinically observed carbapenemases informs on a novel antibiotic resistance mechanism

**DOI:** 10.1074/jbc.RA120.014607

**Published:** 2021-01-13

**Authors:** Kristina M.J. Aertker, H.T. Henry Chan, Christopher T. Lohans, Christopher J. Schofield

**Affiliations:** 1 Chemistry Research Laboratory, University of Oxford, Oxford, United Kingdom; 2 Department of Biomedical and Molecular Sciences, Queen's University, Kingston, Ontario, Canada

**Keywords:** class D serine β-lactamase, antibiotic resistance, carbapenem, β-lactone, carbapenemase, antibiotics, antibiotic action, enzyme mechanism, enzyme kinetics

## Abstract

An important mechanism of resistance to β-lactam antibiotics is via their β-lactamase–catalyzed hydrolysis. Recent work has shown that, in addition to the established hydrolysis products, the reaction of the class D nucleophilic serine β-lactamases (SBLs) with carbapenems also produces β-lactones. We report studies on the factors determining β-lactone formation by class D SBLs. We show that variations in hydrophobic residues at the active site of class D SBLs (*i.e.* Trp^105^, Val^120^, and Leu^158^, using OXA-48 numbering) impact on the relative levels of β-lactones and hydrolysis products formed. Some variants, *i.e.* the OXA-48 V120L and OXA-23 V128L variants, catalyze increased β-lactone formation compared with the WT enzymes. The results of kinetic and product studies reveal that variations of residues other than those directly involved in catalysis, including those arising from clinically observed mutations, can alter the reaction outcome of class D SBL catalysis. NMR studies show that some class D SBL variants catalyze formation of β-lactones from all clinically relevant carbapenems regardless of the presence or absence of a 1β-methyl substituent. Analysis of reported crystal structures for carbapenem-derived acyl-enzyme complexes reveals preferred conformations for hydrolysis and β-lactone formation. The observation of increased β-lactone formation by class D SBL variants, including the clinically observed carbapenemase OXA-48 V120L, supports the proposal that class D SBL-catalyzed rearrangement of β-lactams to β-lactones is important as a resistance mechanism.

The most important identified mechanism of resistance to the clinically important β-lactam–containing antibiotics likely involves their degradation by β-lactamases (1, 2). There are two mechanistic classes of β-lactamases, *i.e.* the serine β-lactamases (SBLs) and the zinc ion–dependent metallo-β-lactamases. The SBLs are presently more important from a clinical perspective and are subdivided into Ambler classes A, C, and D, with the metallo-β-lactamases forming class B (3, 4). Of the SBLs, the highly diverse class D SBLs are of increasing concern. Some class D SBLs efficiently catalyze the degradation of carbapenems, a class of β-lactams often used as last resort drugs because of their breadth of antibacterial activity and resistance to degradation by some SBLs (5, 6, 7, 8).

SBL catalysis proceeds via a hydrolytically labile acyl-enzyme complex (AEC) intermediate, formed by addition–elimination reaction of the nucleophilic serine residue (Ser^70^ in class D SBL OXA-48) with a substrate β-lactam (Fig. 1) (1, 9). An analogous AEC is formed in the case of β-lactam antibiotic-mediated inhibition of their bacterial targets, penicillin-binding proteins. By contrast with penicillin-binding proteins, in the case of SBL catalysis, the AEC undergoes efficient hydrolysis (Fig. 1*A*). Uniquely among studied SBL classes, the class D SBLs employ a carbamylated lysine for general acid/base catalysis (Fig. 1*B*) (10, 11).

**Figure 1:**
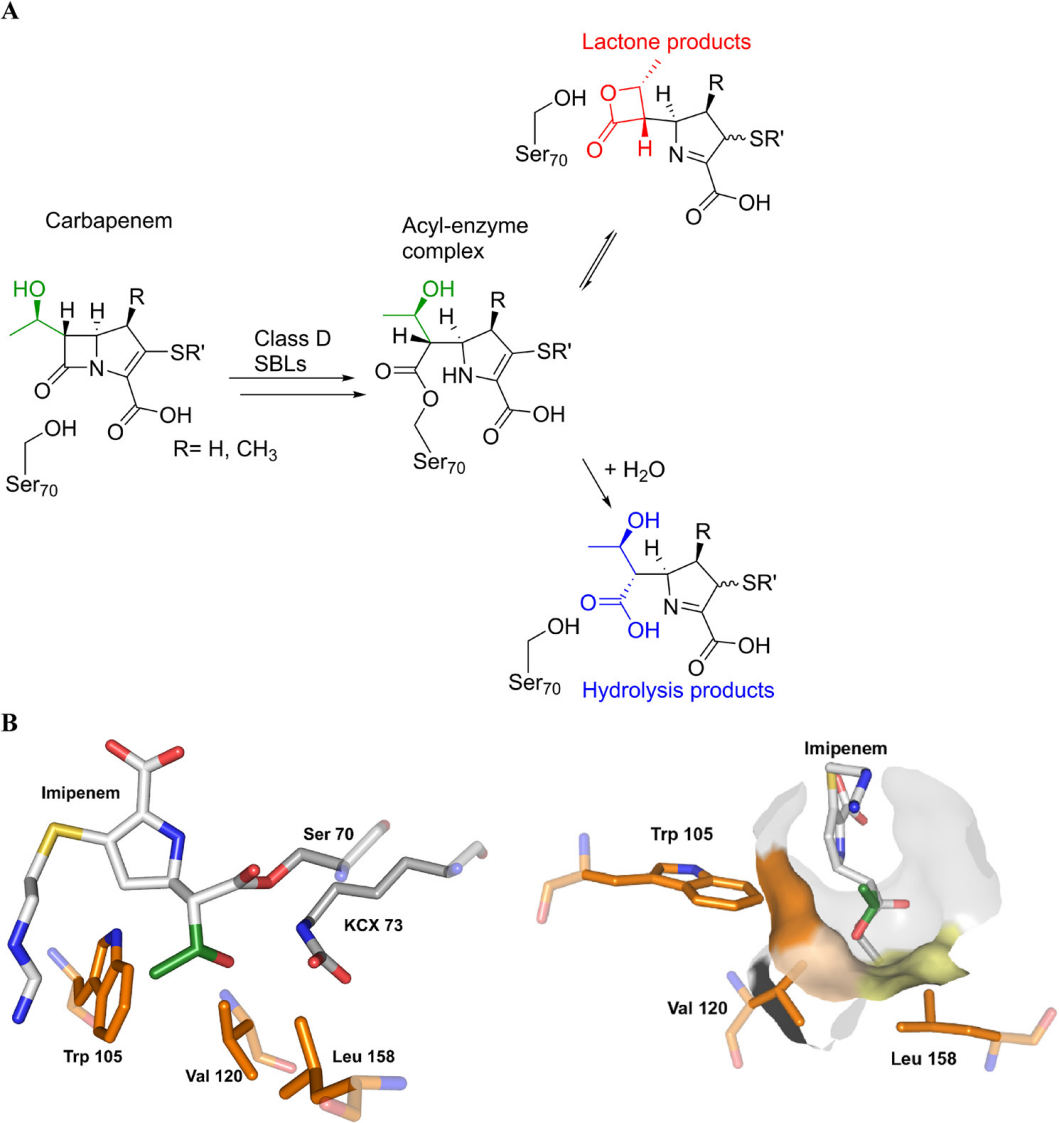
**Reactions of carbapenems with class D serine β-lactamases.***A*, class D SBLs can convert 1β-methyl–substituted carbapenems to reversibly form β-lactone (*red*) and irreversibly form hydrolysis (*blue*) products. The C-6 hydroxyethyl side chain is in *green*. Note that there are different possible tautomeric states of the carbapenem-derived pyrroline ring, both in the AEC and for the products; in the case of both hydrolysis and lactone-forming reactions, the major nascent product is likely the enamine tautomer (Fig. S5) (15). *B*, crystallographically derived views of the active site of OXA-48 after reaction with imipenem (*white*) (PDB entry 5QB4) (18). The imipenem C-6 hydroxyethyl side chain (*green*) is positioned to interact with the side chains of Val^120^, Leu^158^, and Trp^105^ (*orange*). Nucleophilic Ser^70^ (*gray*) and carbamylated Lys^73^ (*KCX 73*; *gray*) are also shown.

All current carbapenem drugs have a C-6 hydroxyethyl side chain. Some carbapenems, *e.g.* imipenem, have a hydrogen substituent in the 1β position, whereas others, *e.g.* meropenem, have a 1β-methyl substituent, which hinders degradation by human renal dehydropeptidase-1 (Fig. 1) (12). The carbapenem C-6 hydroxyethyl side chain is proposed to help stabilize the SBL AEC by slowing its hydrolysis, thereby enabling partial carbapenem resistance to SBL degradation (9). In support of this proposal, crystallographic studies of the AEC formed between the class A SBL TEM-1 and the carbapenem, imipenem, suggest that the C-6 hydroxyethyl side chain interacts with the “hydrolytic” water molecule in the active site (13); it is proposed that this hydrogen-bonding interaction disfavors nucleophilic attack of the water molecule.

The classical mechanism of SBL catalysis has recently been extended in work showing that class D SBLs can isomerize 1β-methyl–substituted carbapenems to form β-lactones, in addition to hydrolysis products (Fig. 1), raising the possibility of nonhydrolytic SBL-mediated carbapenem resistance (14, 15). The β-lactone products are proposed to be formed by initial nucleophilic attack of the C-6 hydroxyethyl hydroxyl group onto the ester carbonyl of the AEC. In support of this mechanism and arguing against their formation via reaction of a noncovalently bound complex, at least in some cases, the β-lactones can react reversibly with class D SBLs to give a covalently modified complex (14). The carbapenem-derived β-lactones are less potent SBL inhibitors than the parent carbapenems, but with optimization to enable them to form stable acyl-enzyme complexes, β-lactones may form the basis of a new type of SBL inhibitor (14).

The factors determining bifurcation between hydrolysis and β-lactone formation are of interest from the perspectives of carbapenem antibiotic resistance and antibacterial drug development. Kinetic analyses show that the class D SBLs degrade 1β-methyl–substituted carbapenems more slowly than those with a 1β-hydrogen substituent, leading to the proposal that β-lactone formation may occur when hydrolysis is disfavored at the AEC (16, 17). Computational studies indicate that the carbapenem 1β-methyl substituent may influence the C-6 hydroxyethyl side chain conformation in a manner favoring β-lactone formation (or, at least, disfavoring hydrolysis) (14). Crystallographic studies have shown that, in the AEC derived from class D SBLs, the carbapenem C-6 hydroxyethyl side chain is positioned in a hydrophobic pocket (18, 19, 20). In OXA-48, one of the most important class D SBLs from a clinical perspective (5), this pocket is formed by the side chains of the highly, but not universally, conserved residues Val^120^, Trp^105^, and Leu^158^ (Fig. 1*B*) (18).

We report studies aimed at investigating the contribution of class D SBL residues involved in binding the carbapenem C-6 hydroxyethyl side chain on the kinetics and the reaction outcome of carbapenem degradation, focusing on the ratio of β-lactone to hydrolysis products formed. The results reveal that the identity of hydrophobic active site residues can affect the balance between carbapenem hydrolysis and isomerization to form β-lactone products. The clinically observed OXA-48 V120L variant (OXA-519) (21), which catalyzes carbapenem degradation more rapidly than the WT OXA-48, substantially favors β-lactone formation over hydrolysis. A similar increase in β-lactone production is found with an analogous OXA-23 variant (OXA-23 V128L) (8). Importantly, in addition to the reported formation of β-lactone products from carbapenems with 1β-methyl substituents, we observed OXA-519 catalyzed formation of β-lactones from imipenem and panipenem, which have a 1β-hydrogen substituent.

## Results

### Steady-state kinetics

The roles of the OXA-48 residues surrounding the carbapenem C-6 hydroxyethyl side chain (*i.e.* Val^120^, Leu^158^, and Trp^105^) were investigated by mostly conservative substitutions, aiming to alter the carbapenem C-6 hydroxyethyl side chain conformation while maintaining catalytic activity and the overall protein fold. Thus, recombinant forms of the OXA-48 V120L, V120I, L158V, L158I, W105A, and W105F variants were produced and highly purified (Fig. S1). Comparison of recombinant WT OXA-48 with these six OXA-48 variants by CD spectroscopy and thermal melting analyses indicates that the substitutions do not have a substantial impact on the overall fold (Fig. S1).

To investigate the impact of the six OXA-48 substitutions on catalytic activity, we initially carried out kinetic analyses monitoring product formation with the cephalosporin nitrocefin and substrate depletion with the two carbapenems meropenem and imipenem (Table 1). The carbapenems employed comprised one with (meropenem) and one without (imipenem) a 1β-methyl substituent. In all cases, the OXA-48 variants were reduced in their catalytic activity for nitrocefin and both carbapenems compared with WT OXA-48, as judged by *k*_cat_/*K_m_* values.

**Table 1:** Steady-state kinetic analyses of wildtype OXA-48 and variants with nitrocefin, meropenem, and imipenem, as determined spectrophotometrically. Assays were performed using nitrocefin (5–1500 μm) and enzyme (25–500 pm) in 50 mm sodium phosphate, pH 7.5, or meropenem (1.75–500 μm), or imipenem (5–500 μm) with enzyme (1.5–400 nm) in 50 mm sodium phosphate, pH 7.5, supplemented with 50 mm sodium bicarbonate. Kinetic parameters are means ± standard deviations (*n* = 3)

OXA-48	Substrate	*k*_cat_	*K_m_*	*k*_cat_/*K_m_*
		*s*^−*1*^	μ*m*	*(m*^−*1*^*s*^−*1*^*)* × *10*^−*6*^
WT	Nitrocefin	593.2 ± 18.3	23.9 ± 3.5	24.8 ± 3.7
	Meropenem	0.087 ± 0.01	<1.9^a^	>0.045^a^
	Imipenem	11.4 ± 0.5	57.7 ± 9.3	0.20 ± 0.03
V120L	Nitrocefin	287.1 ± 11.7	184.1 ± 22.8	1.5 ± 0.20
	Meropenem	1.1 ± 0.08	51.0 ± 11.8	0.021 ± 0.005
	Imipenem	0.49 ± 0.006	6.8 ± 0.6	0.071 ± 0.006
V120I	Nitrocefin	323.2 ± 11.1	40.1 ± 4.9	8.1 ± 1.02
	Meropenem	0.94 ± 0.01	12.6 ± 1.4	0.075 ± 0.008
	Imipenem	17.9 ± 1.4	208.2 ± 45.2	0.086 ± 0.00
W105F	Nitrocefin	178.1 ± 16.7	298.4 ± 37.8	0.60 ± 0.09
	Meropenem	0.038 ± 0.0007	<1.75^a^	>0.022^a^
W105A	Nitrocefin	70.72 ± 1.5	218.1 ± 9.5	0.32 ± 0.02
	Meropenem^b^			
L158V	Nitrocefin	54.9 ± 4.3	16.1 ± 3.3	3.4 ± 0.8
	Meropenem	0.0081 ± 0.0002	<2^a^	>0.0041^a^
L158I	Nitrocefin	59.8 ± 3.5	8.1 ± 2.2	7.4 ± 2.1
	Meropenem	0.031 ± 0.005	7.1 ± 4.1	0.0044 ± 0.003


a The *K_m_* values shown are the lowest [S] at which turnover could be accurately measured. The actual *K_m_* values are thus lower and the *k*_cat_/*K_m_* values are larger than those given.

b Not determined because of slow reaction rate.

For all the OXA-48 variants, the results imply nitrocefin is a better substrate than the two carbapenems, as judged by *k*_cat_ and *k*_cat_/*K_m_* values, although in some cases the *K_m_* values for the carbapenems are lower than for nitrocefin (Table 1). With nitrocefin, the *K_m_* values are increased for OXA-48 V120L, V120I, W105F, and W105A but are decreased for the L158V and L158I variants. Meropenem degradation by OXA-48 W105A was too slow for kinetic constants to be accurately determined. Mass spectrometric experiments suggest that the acylation of OXA-48 W105A by meropenem occurs efficiently (Fig. S2), implying that this substitution may disfavor deacylation of the AEC intermediate.

Comparison of the results for the two carbapenems with the OXA-48 V120L and V120I variants reveals interesting variations in the *k*_cat_ and *K_m_* values relative to WT OXA-48. The *k*_cat_ values increase for meropenem with both the V120L and V120I variants and increases for imipenem with V120I variant but decreases for imipenem with the V120L variant. Compared with WT OXA-48, both the V120L and V120I variants catalyze meropenem degradation less efficiently than imipenem (as judged by *k*_cat_/*K_m_* values). These results reveal that interactions between specific active site residues and specific carbapenems can impact on kinetics; we hence envisaged that such interactions might alter the ratio of hydrolysis to β-lactone products.

To investigate whether lysine carbamylation is substantially altered in the variants compared with WT OXA-48, WT OXA-48 and the V120L and V120I variants were treated with NaH^13^CO_3_, and the extent of carbamylation was monitored by ^13^C NMR (Fig. S3) (11). These results suggest that the carbamylation state of all three enzymes is approximately comparable. With added sodium bicarbonate, the *k*_cat_ and *K_m_* values with nitrocefin for WT OXA-48 and the V120L and V120I variants all increased (Table S1).

To explore the generality of these observations, the impact of substituting Val^128^ (equivalent to Val^120^ in OXA-48) on the activity of OXA-23, a clinically important class D SBL that shares 39% sequence identity with OXA-48, was also examined. The V128L and V128I variants of OXA-23 were produced and highly purified (Fig. S4) (22, 23). With nitrocefin, the *k*_cat_/*K_m_* values for OXA-23 V128I and V128L are lower than for WT OXA-23, because the *K_m_* values are substantially higher for both variants. Imipenem is a better substrate than meropenem for WT OXA-23 and, to a lesser extent, for OXA-23 V128I based on *k*_cat_/*K_m_* values. However, imipenem and meropenem are similarly good substrates for OXA-23 V128L (Table S2).

### NMR turnover assays

The overall kinetic analyses with the class D SBL variants reveal variations in both *k*_cat_ and *K_m_* values for the three tested substrates. To investigate whether these variations correlate with the ratio of hydrolysis:β-lactone products formed, we carried out studies using ^1^H NMR (600 MHz) spectroscopy. This enables differentiation between the two product types, which is not possible when using standard UV-visible assays for carbapenem turnover (14).

We monitored product formation using the signal corresponding to the C-9 methyl group, which manifests distinctive resonances for the carbapenem starting material, the hydrolysis products, and the β-lactone products (1.22, 1.18, and 1.55 ppm, respectively) (14, 15). It should be noted that, in principle, the hydrolysis and β-lactone products can be produced in two epimeric 1-pyrroline forms (*i.e.* (*S*)-Δ^1^ imine and (*R*)-Δ^1^ imine), as well as the tautomeric 2-pyrroline (Δ^2^ enamine) form (Fig. S5A). Recent work has indicated that the Δ^2^ enamine is likely the predominant nascent enzymatic product with most class D SBLs, including OXA-48 and OXA-23 (15). In the case of the products derived from 1β-methyl–substituted carbapenems, rapid tautomerization yields the (*R*)-Δ^1^ imine product as the kinetic imine product (which is likely, at least predominantly, a nonenzymatic product) with subsequent conversion to the more thermodynamically stable (*S*)-Δ^1^ imine product (Fig. S5B) (15). Under our standard NMR assay conditions with the 1β-methyl–substituted carbapenem meropenem, the (*S*)-Δ^1^ imine product was usually the major product form observed for both the hydrolysis and β-lactone products, within the time frames of our assays (Fig. S5).

**Figure 2:**
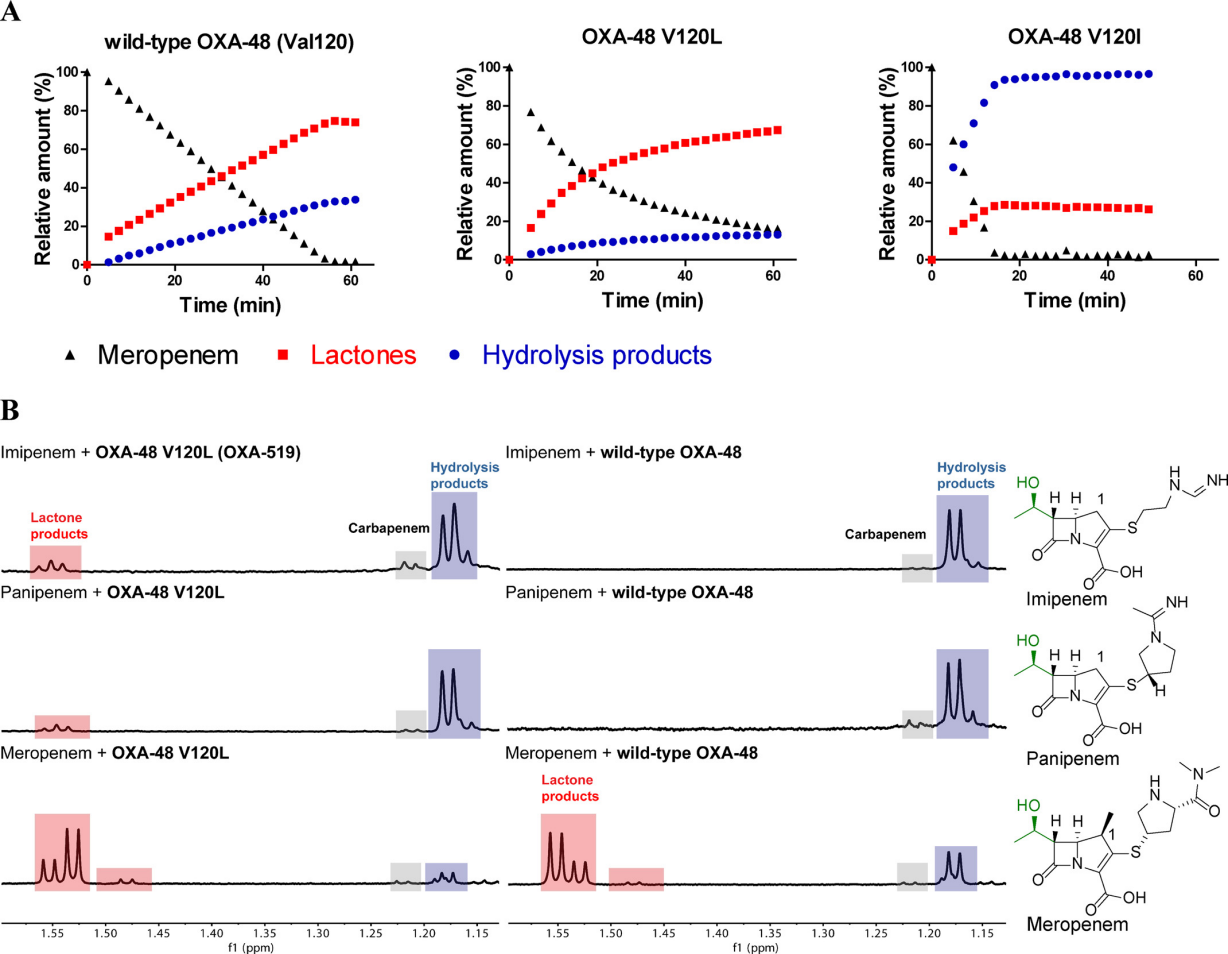
**The identity of the hydrophobic residue at position 120 impacts on the extent of β-lactone formation.***A*, time-course analyses showing the products formed from meropenem in the presence of WT OXA-48, OXA-48 V120L (OXA-519), and OXA-48 V120I, as monitored by ^1^H NMR (600 MHz). *t* = 0 refers to measurements made prior to enzyme addition. *B*, comparative analysis of β-lactones and hydrolysis products derived from imipenem, panipenem, and meropenem formed by OXA-48 V120L and WT OXA-48, measured by ^1^H NMR spectroscopy (600 MHz). OXA-519 was observed to form β-lactones from all three tested carbapenems, whereas WT OXA-48 was only observed to produce β-lactones from the 1β-methyl–substituted carbapenem (meropenem). Note that the depletion of β-lactone levels at long time points is expected because the β-lactones bind reversibly with the class D SBLs and can undergo hydrolysis (14).

All the studied OXA-48 variants catalyze reaction of the 1β-methyl–substituted carbapenem, meropenem to form both β-lactone and hydrolysis products (Fig. 2*A* and Fig. S6). The OXA-48 V120I, W105F, L158V, and L158I variants produce more of the hydrolysis products than the β-lactones. Of all the tested enzymes, the V120I variant forms the lowest amount of β-lactone relative to hydrolysis products. Although the WT and W105A enzymes produce a small excess of β-lactone, the V120L variant, which is clinically observed (21), produces considerably more β-lactones than hydrolysis products. Testing of the OXA-48 V120L variant with other 1β-methyl–substituted carbapenems (ertapenem and biapenem) similarly shows that β-lactone formation is the predominant pathway under the tested conditions (Fig. S7). These observations reveal that substitutions other than those directly involved in the acid/base catalytic machinery can influence the ratio of hydrolysis to β-lactone formation.

Previous experiments have indicated that WT OXA-48 does not form β-lactones from carbapenems with a 1β-hydrogen rather than a 1β-methyl substituent, such as in the clinically used drugs imipenem and panipenem (16, 17). However, upon analyzing the products of the reactions of OXA-48 V120L with imipenem and panipenem, we observed new products in the ^1^H NMR spectra that are not observed with WT OXA-48 (Fig. 2*B* and Fig. S6). Characterization of these products by NMR analyses reveals formation of the previously unobserved β-lactones derived from imipenem and panipenem, in addition to the established hydrolysis products (Tables S3 and S4 and Figs. S8–S15). This observation demonstrates that β-lactone formation by class D SBLs is not limited to 1β-methyl–substituted carbapenems.

We then investigated whether the identity of the residue at position 120 (OXA-48 numbering) plays a role in determining the reaction outcome for other class D SBLs. With meropenem, the OXA-23 V128L variant was observed to favor β-lactone formation compared with WT OXA-23, similar to what was observed for WT OXA-48 and OXA-48 V120L (Fig. S16). As for the observations with OXA-48 V120L, OXA-23 V128L forms β-lactone products from imipenem and panipenem, both of which lack a 1β-methyl substituent (Fig. S17). However, the OXA-23 V128I variant does not favor hydrolysis over β-lactone formation in the manner that was observed for OXA-48 V120I (Fig. 2*A* and Fig. S16).

Comparison of the ratios of hydrolysis:β-lactone products (Fig. 2*A* and Figs. S6, S7, S16, and S17) with the kinetic data for *k*_cat_, *K_m_*, and *k*_cat_/*K_m_* values (Table 1 and Table S2) for the different tested class D SBL variants does not reveal any clear relationships. This implies that the ratio of hydrolysis:β-lactone products is not simply related to binding efficiency or stability of the AEC but rather is a function of specific interactions between carbapenem-derived intermediates and the active site.

### Conformations of the C-6 hydroxyethyl side chain observed by crystallography

**Figure 3:**
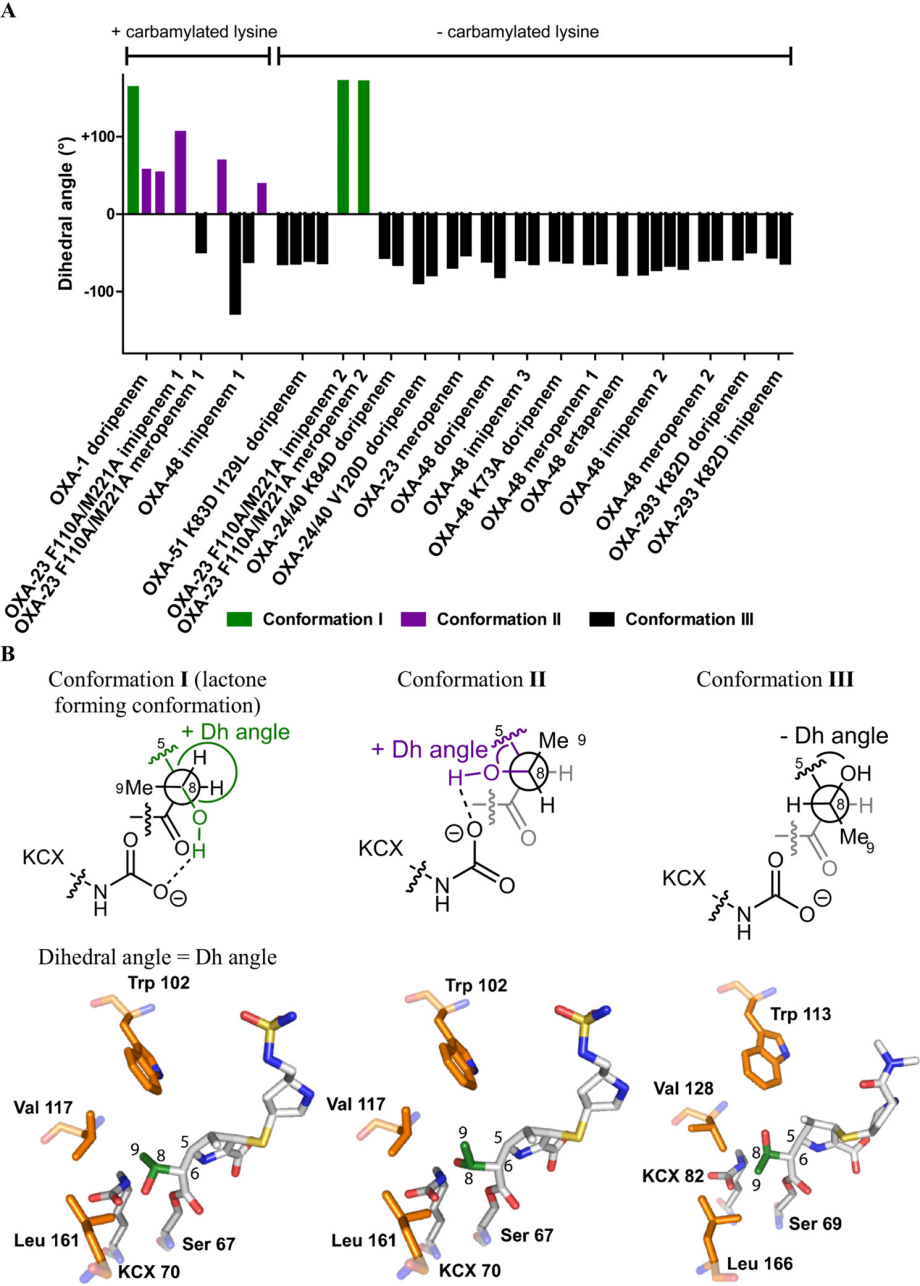
**Crystallographically observed conformations of the carbapenem C-6 hydroxyethyl side chain.***A*, dihedral angles (*Dh angles*) of the hydroxyethyl side chain (O C-8 C-6 C-5) in the AECs of class D SBLs and carbapenems, in the presence and absence of a carbamylated lysine. The following structures were analyzed: OXA-1 with doripenem, pH 7.5 (PDB entry 3ISG) (19); OXA-23 F110A, M221A with imipenem, pH 7.0 (PDB entry 6N6X) (20); OXA-23 F110A, M221A with meropenem, pH 7.0 (PDB entry 6N6Y) (20); OXA-23 F110A, M221A with meropenem, pH 4.1 (PDB entry 6N6V) (20); OXA-23 F110A, M221A pH 4.1 with imipenem, (PDB entry 6N6U) (20); OXA-24/40 K84D with doripenem, pH 8.5 (PDB entry 3PAE) (24); OXA-24/40 V130D with doripenem, pH 8.5 (PDB entry 3PAG) (24); OXA-23 with meropenem, pH 4.1 (PDB entry 4JF4) (25); OXA-51 K83D, I129L with doripenem, pH 6.5 (PDB entry 5L2F) (26); OXA-13 with imipenem (PDB entry 1H5X) (28); OXA-48 with imipenem, pH 7.5 (1) (PDB entry 5QB4) (18); OXA-48 K73A with doripenem, pH 4.0 (PDB entry 6PXX) (30); OXA-48 with ertapenem, pH 4.0 (PDB entry 6P99) (32); OXA-48 with imipenem, pH 4.0 (3) (PDB entry 6P97) (32); OXA-48 with meropenem, pH 4.0 (1) (PDB entry 6P98) (32); OXA-48 with doripenem, pH 4.0 (PDB entry 6P9C) (32); OXA-48 with imipenem, pH 4.6 (2) (PDB entry 6PTU); OXA-48 with meropenem, pH 4.6 (2) (PDB entry 6PT1); OXA-239 K82D with doripenem, pH 4.2 (PDB entry 5WI7) (27); and OXA-239 K82D with imipenem, pH 4.2 (PDB entry 5WIB) (27). *Numbers* are used in cases in which there were more than one of the same enzyme: carba penem crystal structure. This analysis suggests that the carbamylated lysine is an important determinant of the C-6 hydroxyethyl side chain conformation. *B*, view of hydroxyethyl conformations in the OXA-1 active site with doripenem (conformations I and II) and the OXA-23 F110A/M221A active site with meropenem (conformation III), showing dihedral angles and interactions of the carbamylated lysine and surrounding residues with the C-6 hydroxyethyl side chain. Conformation I is proposed to most closely represent the conformation required for β-lactone formation, because the C-6 hydroxyethyl hydroxyl group is positioned in a suitable orientation (*i.e.* has a favorable Bürgi–Dunitz trajectory) relative to the AEC carbonyl. Note, Trp^113^/Val^128^/Leu^166^ in OXA-23 and Trp^102^/Val^117^/Leu^161^ in OXA-1 are equivalent to Trp^105^/Val^120^/Leu^158^ in OXA-48. *KCX 70*, carbamylated Lys^70^.

To investigate the structural basis of the observed β-lactone *versus* hydrolysis product ratios, we analyzed crystallographically observed AECs derived from the reactions of class D SBLs with carbapenems (PDB entry 1H5X) (18, 19, 20, 24, 25, 26, 27, 28, 29). The analyses reveal three different conformations (conformations I, II, and III) that are adopted by the C-6 hydroxyethyl side chain in the AECs (Fig. 3, *A–C*). Note that some of the crystal structures do not have a carbamylated lysine, possibly because of the low pH used for crystallization or the substitution of the lysine with another residue to generate a deacylation-deficient enzyme. Further, it is possible that the crystallographically observed conformations do not reflect those present during efficient catalysis.

In conformation I, as observed in a crystal structure of OXA-1 with doripenem (19), the C-9 methyl group of the C-6 hydroxyethyl side chain is oriented toward Val^117^ of OXA-1 (corresponding to Val^120^ in OXA-48 and Val^128^ in OXA-23), a key position with respect to β-lactone formation as shown in our work. The C-6 hydroxyethyl hydroxyl group is positioned between the carbamylated lysine and the ester carbonyl group of the AEC. Hence, the hydroxyl group is apparently positioned for favorable reaction with the AEC carbonyl group, according to Bürgi–Dunitz analysis (Fig. S18) (30), leading to β-lactone formation. It seems likely that this “β-lactone–forming conformation” will also block the entry of water into a productive position for hydrolysis of the AEC.

In conformation II, the C-6 hydroxyethyl hydroxyl group is oriented toward Val^117^ of OXA-1 (corresponding to Val^120^ in OXA-48 and Val^128^ in OXA-23), and the C-8 hydrogen of the C-6 hydroxyethyl side chain is oriented toward the anticipated position of the hydrolytic water during reaction of the AEC (Fig. 3). Of the three crystallographically observed conformations, conformation II is thus likely closest to that relevant for productive hydrolysis of the AEC.

Conformation III is observed in most (∼93%) of the AEC structures lacking lysine carbamylation (Fig. 3) (25, 31). However, there are recently reported AEC structures arising from the reaction of OXA-10 with ertapenem and imipenem and of OXA-10 V117L with meropenem that manifest conformation III in the presence of a carbamylated active site lysine (PDB entry 6SKQ). The uncarbamylated structures are not expected to represent catalytically active complexes, because lysine carbamylation is critical for both hydrolysis and β-lactone formation by class D SBLs (24, 29). In conformation III, the C-6 hydroxyethyl side chain is oriented such that its hydroxyl group is directed toward the side chain of Trp^113^ in OXA-23 (Trp^105^ in OXA-48 and Trp^102^ in OXA-1), *i.e.* toward a hydrophobic pocket and away from the AEC carbonyl group. The C-9 methyl group of the C-6 hydroxyethyl side chain is located between the carbamylated lysine and the carbonyl group of the AEC, apparently in a manner that would hinder productive approach of the water molecule required for AEC hydrolysis. Thus, conformation III appears to be unfavorable for both hydrolysis and β-lactone formation.

Overall, the analyses of class D SBL-carbapenem–derived AEC crystal structures imply that conformation I best resembles the conformation required for β-lactone formation, whereas conformation II best reflects that leading to AEC hydrolysis. The different effects of the tested class D SBL variants on hydrolysis:β-lactone product ratios, in particular those caused by residue 120 (OXA-48 numbering), likely arise in part from biases in the ratio of conformations I and II during catalysis.

### Bioinformatic analyses

To investigate the potential clinical relevance of β-lactone formation by class D SBLs, the sequences of all class D SBL sequences in the Beta-Lactamase DataBase (32) were aligned, and the identities of the amino acid residues at position 120 (OXA-48 numbering) were analyzed (Fig. S19). The resultant analysis shows that, at present, ∼2% of all reported class D SBL sequences contain a Val-to-Leu substitution at residue 120 (OXA-48 numbering). This substitution is found in eight different class D SBL subfamilies: OXA-51, OXA-62, OXA-153, OXA-157, OXA-156, OXA-493, OXA-48, and OXA-10. ∼39% of class D SBLs have a Val-to-Ile substitution at residue 120 (OXA-48 numbering); this substitution is found in three class D SBL subfamilies: OXA-51, OXA-213, and OXA-12. The amino acid sequence of the naturally occurring class D SBL OXA-519 is identical to that of OXA-48 V120L (21). A similar Val-to-Leu substitution has been identified in the OXA-10 variant OXA-655 (in which an additional T26M substitution did not impact on catalytic activity) (33). Both OXA-519 and OXA-655 show enhanced carbapenemase activity relative to their parent class D SBLs, with minimum inhibitory concentration values for *Escherichia coli* strains expressing these enzymes revealing reduced carbapenem susceptibility (21, 33). Thus, it is possible that increased carbapenem resistance mediated by these class D SBL variants relates, at least in part to increased β-lactone formation.

## Discussion

Because of their ability to confer resistance to carbapenems, which are often used as antibiotics when other drugs have failed, the class D SBLs are of considerable clinical relevance. Although the early identified class D SBLs appeared to have a rather narrow substrate specificity in which they hydrolyzed oxacillin in preference to other β-lactam antibiotics, the number of class D SBLs has escalated rapidly in recent years, with more than 930 variants now reported, many with broad substrate profiles (32). From a mechanistic perspective, their use of a carbamylated lysine for general acid/base catalysis means they are unique among β-lactamases. As a consequence, the class D SBLs have been the subject of many mechanistic and biophysical analyses. The recent finding that the class D SBLs react with 1β-methyl–substituted carbapenems to reversibly produce substantial quantities of isomeric β-lactones, in addition to the established hydrolysis products, was therefore unexpected (14). The ability of β-lactam–derived AECs to undergo nonhydrolytic inhibitor side chain–mediated fragmentation is well-precedented; for example, the AECs derived from certain penicillins with isolated transpeptidases can undergo nonhydrolytic fragmentation (34). However, class D SBL-catalyzed β-lactone formation is striking because of its apparent functional relevance, *i.e.* it is proposed to represent a new mechanism of resistance to β-lactam antibiotics involving isomerization to give a less active or inactive product rather than the established mechanism leading to hydrolysis products.

In our initial report on β-lactone formation by class D SBLs (*i.e.* OXA-10, OXA-23, and OXA-48), we only observed β-lactone formation with 1β-methyl–substituted carbapenems, such as meropenem and ertapenem (14). One clinically relevant outcome of the current study is that certain class D SBL variants, *i.e.* OXA-48 V120L (OXA-519) and OXA-23 V128L, can catalyze β-lactone formation from widely used carbapenems lacking a 1β-methyl substituent, *e.g.* panipenem or imipenem.

Given that hydrolytic deacylation of the AEC is reported to be rate-limiting (at least for some class D SBLs, *e.g.* OXA-10) (35), it is reasonable to propose that factors disfavoring hydrolysis may increase the rate of β-lactone formation. Although this might be a factor, the evidence presented here coupled with the analysis of the previously reported crystal structures implies that subtle interactions at the active site, involving hydrophobic residues that affect the carbapenem C-6 hydroxyethyl side chain conformation, can alter the ratio of hydrolysis: β-lactone products and indeed whether or not β-lactones are formed at all.

A clear example of this is the case of residue 120 (OXA-48 numbering), the side chain of which is located in a hydrophobic pocket that contributes to the stability of the catalytically important carbamylated lysine (35). When Val^130^ and Val^117^ (equivalent to Val^120^ in OXA-48) were substituted to aspartic acid in OXA-24/40 or OXA-1, respectively, deacylation-deficient enzymes are produced in a manner proposed to be due to impaired carbamylation (24, 36). Our results reveal that the more conservative Val-to-Leu and Val-to-Ile substitutions maintain lysine carbamylation and enzyme catalytic activity (Fig. S3 and Table 1). OXA-48 V120I favors hydrolysis over β-lactone formation; indeed, of all the tested enzymes, the OXA-48 V120I variant formed the lowest amount of β-lactones relative to hydrolysis products (Fig. 2*A*). By contrast, WT OXA-48 and the OXA-48 V120L variant favor β-lactone production over hydrolysis with 1β-methyl–substituted carbapenems (meropenem, ertapenem, and biapenem) (Fig. S7). Further, with imipenem and panipenem, both of which lack a 1β-methyl substituent, OXA-48 V120L produces β-lactone products, albeit at a lower relative level than observed with 1β-methyl–substituted carbapenems. The results for the OXA-48 Val^120^ substitutions were mirrored with studies on OXA-23 Val^128^ substitutions, showing that the results are not limited to a single class D SBL subfamily.

The results for Val^120^ contrast with those observed for similar substitutions with Leu^158^ of OXA-48. OXA-48 L158I and L158V both manifest decreased carbapenemase activity and produce less β-lactone, indicating that the intermediate conformation required for β-lactone formation is disfavored, at least relative to that for hydrolysis (Fig. 3). Leu^158^ has been proposed to act as a “water-gate residue,” enabling productive access of a hydrolytic water to the class D SBLs active site (25). It is possible that the β-branched side chains of isoleucine and valine hinder catalytically productive access of hydrolytic water to the active site, thus stabilizing the AEC. If so, this does not occur in a manner that favors β-lactone formation, supporting the proposal that simply stabilizing the AEC with respect to hydrolysis is insufficient to enable/promote β-lactone formation.

This proposal is further validated by work on Trp^105^ variants. The active sites of most class D SBLs are proposed to have a hydrophobic bridge, involving their α3–α4 and β6–β7 loops, that is involved in substrate/intermediate(s) interactions (20). Although OXA-48 may not have an analogous bridge (37), the OXA-48 α3–α4 loop that bears Trp^105^ is likely involved in substrate/intermediate binding. Neither of the OXA-48 W105F or W105A variants manifested increased β-lactone formation (Fig. S6), although OXA-48 W105A is defective in the deacylation step (Table 1 and Fig. S2).

Most of our kinetic data, as well as those previously reported (17), show that carbapenems with a 1β-methyl substituent are degraded substantially more slowly than those without such a substituent. In a previous report (14), we proposed, in part based on calculations involving noncovalent interactions, that the presence of a carbapenem 1β-methyl substituent limits the conformational flexibility of the C-6 hydroxyethyl side chain at the AEC stage in a manner promoting β-lactone formation over hydrolysis. OXA-48 V120L and OXA-23 V128L may overcome this reduced enzyme activity with carbapenems with a 1β-methyl substituent by sterically promoting a conformation favoring β-lactone formation.

Given the very large number of class D SBL variants and the apparent ability of class D SBLs to evolve new activities, it would seem very possible that class D SBL variants other than those studied by us have β-lactone–forming capabilities. Even if this is not presently the case, given the selection pressure exerted by antibiotic use, it is likely that other class D SBLs have evolved the capability to rearrange β-lactams to give β-lactones, and possibly, other novel ways to fragment AECs.

The question also arises as to why β-lactone formation has not been observed in other classes of SBLs. It cannot be ruled out but would seem unlikely, at least for well-studied SBLs, such as the class A SBLs. In the class A SBLs, the residues that interact with the carbapenem C-6 hydroxyethyl side chain differ significantly from those in the class D SBLs. Instead of the valine residue (*e.g.* Val^120^ in OXA-48), a highly conserved asparagine residue (*e.g.* Asn^132^ in GES-5) is found in an approximately analogous position, whereas a glutamic acid (*e.g.* Glu^166^ in GES-5) acts as a general acid/base during catalysis rather than the carbamylated lysine of the class D SBLs (38). Hydrogen bonding between the hydroxyl group of the C-6 hydroxyethyl side chain with this highly conserved asparagine residue may limit the conformational flexibility of the hydroxyethyl side chain, thereby disfavoring β-lactone formation. Whether or not class A/C SBLs have evolved, or may evolve, the capacity to catalyze AEC fission via β-lactone formation or other nonhydrolytic reactions (*e.g.* oxazolone formation from penicillins/cephalosporins with a nucleophilic acetamido side chain) is an open question. Evidence of the *in vivo*/clinical relevance of β-lactone formation is of interest, and this is something that we are pursuing in ongoing work including with clinically relevant strains, harboring relevant OXA variants (21, 33, 39).

## Experimental procedures

### Antibiotics

Biapenem and ertapenem were from Glentham Life Sciences; meropenem and imipenem were from Molekula; and panipenem was from Ontario Chemical Inc.

### Protein production and purification

The pNIC28-Bsa4 plasmids encoding most OXA-48 variants (L158I, L158V, V120I, and V120L) and the pOPINF plasmids encoding OXA-23 variants (V128I and V128L) were prepared by the QuikChange site-directed mutagenesis protocol. The reaction mixture consisted of 11.3 μl of sterile deionized water, 5 μl of 5× Q5 reaction buffer, 5 μl of 5× high GC enhancer, 1.25 μl each of 10 μm forward and reverse oligonucleotide primers (designed using the QuikChange site-directed mutagenesis protocol), 1 μl of template plasmid, 0.5 μl of 10 mm dNTP mix, and 0.5 μl of Q5 high-fidelity DNA polymerase (NEB, 2000 units ml^−1^). The following thermocycling conditions were applied: an initial 2 min at 94 °C; then 20 cycles of denaturing step 45 s at 94 °C, annealing step 60 s at 55–67 °C (depending on primer melting temperatures), and extension step 20 min at 68 °C; and a final step of 20 min at 68 °C. To digest the template, 2 μl of restriction enzyme DpnI (NEB, 20,000 units ml^−1^) was added, and the mixture was incubated at room temperature for 4 h. The mutagenesis reaction product was transformed into *E. coli* XL 10 Gold competent cells (Agilent) and plated onto LB plates supplemented with the appropriate antibiotic. The pNIC28-Bsa4 plasmids encoding for OXA-48 W105A and W105F were prepared using the Q5 site-directed mutagenesis kit protocol (NEB) with Q5 high-fidelity DNA polymerase (NEB, 2000 units ml^−1^), 5× Q5 reaction buffer, and 5× high GC enhancer according to the manufacturer's instructions. The OXA-48 and OXA-23 variants were produced and purified as reported (22, 23). In brief, the cells were lysed by sonication in HisTrap buffer A (50 mm HEPES, pH 7.5, 500 mm NaCl, 20 mm imidazole) with DNase I (Roche) and protease inhibitor (Roche). The OXA-48 and OXA-23 variants were purified by affinity chromatography using a HisTrap column (GE Healthcare) with HisTrap buffer A and HisTrap buffer B (50 mm HEPES, pH 7.5, 500 mm NaCl, 500 mm imidazole) and then by gel filtration column using a Superdex 200 column (GE Life Sciences) with 50 mm sodium phosphate, pH 7.5.

### NMR spectroscopy

For NMR experiments, a Bruker Avance III 700 MHz spectrometer equipped with a TCI inverse cryoprobe and a Bruker AVIII HD 600 MHz spectrometer equipped with a BB-F/^1^H Prodigy N_2_ cryoprobe were used (40). All experiments were conducted at 298 K. Presaturation or excitation sculpting with perfect echo was applied for water suppression. All β-lactamase activity assays were prepared in 50 mm sodium phosphate, pH 7.5, 10% D_2_O with 1 mm substrate and the indicated concentration of enzyme (0.5–100 μm). For the ^13^C-carbamylation experiments, samples contained 700 μm enzyme in 50 mm sodium phosphate, pH 7.5, 10 mm NaH^13^CO_3_ (Sigma–Aldrich) with 10% D_2_O and 0.1 mm EDTA (Sigma–Aldrich). Chemical shifts for imipenem-derived β-lactone and panipenem-derived β-lactone were assigned based on ^1^H, COSY, total correlation spectroscopy, heteronuclear single quantum coherence, spectroscopy and heteronuclear multiple bond correlation spectroscopy.

### MS

Protein samples (1–5 µM) were prepared in 50 mm sodium phosphate, pH 7.5. Intact protein mass spectra were acquired using a Waters Micromass LCT Premier XE spectrometer coupled with an Acquity UPLC system. The LC method employed a gradient of 5–100% acetonitrile in purified water supplemented with 0.1% (v/v) formic acid over 8 min. The data were analyzed using MassLynx version 4.0. To investigate the interaction between OXA-48 W105A and meropenem, 1 μm OXA-48 W105A in 50 mm phosphate, pH 7.5, was mixed with 100 μm meropenem. The reaction was analyzed by electrospray ionization–MS in the positive ion mode using an integrated autosampler/solid-phase extraction RapidFire365 system (Agilent Technologies) coupled to an Agilent 6550 Accurate Mass QTOF mass spectrometer. 50 μl of sample was loaded onto a C4 solid-phase extraction cartridge (Agilent Technologies), and salts were removed by washing the cartridge with 0.1% (v/v) formic acid in water, followed by elution with 85% acetonitrile, 15% water containing 0.1% (v/v) formic acid. The data were analyzed using MassHunter qualitative analysis software version 7 (Agilent Technologies).

### CD spectroscopy

CD spectra were acquired at 23 °C using a Chirascan CD spectrometer (Applied Photophysics model) equipped with a Peltier temperature-controlled cell holder (41). Enzymes were prepared at 0.2 mg ml^−1^ in 10 mm sodium phosphate, pH 7.5, and measured in a 0.1-cm-pathlength cuvette. A wavelength range of 185–260 nm was measured with 0.5-nm increments; each data point was averaged for 1 s. CD spectra were baseline-corrected and smoothed using the Savitzky–Golay filter. Thermal melting temperature measurements using a Chirascan CD spectrometer were obtained at 222 nm, with data points acquired every 1 °C from 20 to 85 °C. Melting curves were fitted to a Boltzmann sigmoidal curve using Prism 5 (GraphPad).

### Enzyme kinetics

Steady-state kinetic reactions were carried out in 100 mm sodium phosphate, pH 7.5, 0.01% Triton X-100 at 25 °C with 96-well UV-Star microplates (Greiner Bio-One). The assays were conducted in at least triplicate measurements (*n* ≥ 3) and were monitored using a PHERAstar FS microplate reader (BMG Labtech) as reported (41). Absorbance changes were monitored at 486 nm for nitrocefin (ε = 20,500 m^−1^ cm^−1^) (42), at 295 nm for meropenem (ε = 10,940 m^−1^ cm^−1^) (43), and at 295 nm for imipenem (ε = 9000 m^−1^ cm^−1^) (43). For each experiment, the enzyme concentration was first optimized. For nitrocefin turnover assays, the following enzyme concentrations were used: WT OXA-48 (25 pm), OXA-48 V120I (25 pm), OXA-48 V120L (50 pm), OXA-48 L158V (500 pm), OXA-48 L158I (500 pm), OXA-48 W105F (500 pm), OXA-48 W105A (250 pm), WT OXA-23 (100 pm), OXA-23 V128I (700 pm), and OXA-23 V128L (500 pm). For meropenem assays, enzyme concentrations of WT OXA-48 (75 nm), OXA-48 V120I (100 nm), OXA-48 V120L (300 nm), OXA-48 L158V (1150 nm), OXA-48 L158I (400 nm), OXA-48 W105F (100 nm), WT OXA-23 (350 nm), OXA-23 V128I (290 nm), and OXA-23 V128L (320 nm) were used. For imipenem assays, enzyme concentrations of WT OXA-48 (1.5 nm), OXA-48 V120I (2 nm), OXA-48 V120L (50 nm), WT OXA-23 (42.5 nm), OXA-23 V128I (50 nm), and OXA-23 V128L (170 nm) were used. For carbapenem assays, 50 mm sodium bicarbonate (Sigma–Aldrich) was added to the assay buffer. Prism 5 (GraphPad) was used for nonlinear regression analysis to obtain *K_m_*, *k*_cat_, and *k*_cat_/*K_m_* kinetic parameters.

### Bioinformatic analyses

The Beta-Lactamase DataBase (32) was used to analyze the residues present at position 120 (OXA-48 numbering) in all reported class D SBLs. Excel (Microsoft Office) was used to extract the residue in this position from the 932 class D SBLs.

## Data availability

All the data are contained within the article.

10.13039/100004440Wellcome Trust (Wellcome) (218246/Z/19/Z) to Christopher J. Schofield
10.13039/100004440Wellcome Trust (Wellcome) (106244/Z/14/Z) to Christopher J. Schofield
10.13039/501100000266UKRI | Engineering and Physical Sciences Research Council (EPSRC) (EP/L016044/1) to Kristina M. J. Aertker
