# Supplementary material for: Analysis of β-lactone formation by clinically observed carbapenemases informs on a novel antibiotic resistance mechanism
Source: J Biol Chem. 2021 Jan 13;295(49):16604–13. doi: 10.1074/jbc.RA120.014607 (PMC7864059; doi:10.1074/jbc.RA120.014607)
Supplement: Supplementary file 1 [file mmc1.pdf]

Supporting Information

Analysis of β-lactone formation by clinically observed carbapenemases informs on a novel antibiotic resistance mechanism

Kristina M. J. Aertker[1], H. T. Henry Chan[1], Christopher T. Lohans[1,2]*, and Christopher J. Schofield[1]*

[1]Chemistry Research Laboratory, University of Oxford, 12 Mansfield Road, Oxford, OX1 3TA, United Kingdom. [2]Department of Biomedical and Molecular Sciences, Queen's University, Kingston, ON, K7L 3N6, Canada.

*To whom correspondence may be addressed: Christopher J. Schofield, Chemistry Research Laboratory, University of Oxford, 12 Mansfield Road, OX1 3TA, Oxford, United Kingdom. Tel.: +44-1865-275625; Email: christopher.schofield@chem.ox.ac.uk
or Christopher T. Lohans, Department of Biomedical and Molecular Sciences, Queen's University, Botterell Hall, 18 Stuart Street Kingston, ON, Canada K7L 3N6, Tel: +1-613-533-2989, Email: christopher.lohans@queensu.ca

Supporting Information Contents

Figure S1. Characterization of wild-type OXA-48 and variants.	S-3
Figure S2. Mass spectra for OXA-48 W105A with meropenem.	S-4
Figure S3. ^{13}C-NMR (150 MHz) spectra supporting lysine carbamylation of the OXA-48 V120I and V120L variants.	S-5
Table S1. Steady-state kinetics of wild-type OXA-48 and variants with nitrocefin in the presence and absence of bicarbonate.	S-5
Figure S4. Mass spectrometric analysis of the N-terminally His_6-tagged OXA-23 variants.	S-6
Table S2. Summary of steady-state kinetic parameters for wild-type OXA-23 and variants with nitrocefin, meropenem, and imipenem.	S-6
Figure S5. Carbapenem derived products formed by class D SBL catalysis.	S-7-8
Figure S6. Product profiles for the OXA-48 W105A, W105F, L158I, and L158V variants with meropenem.	S-9
Figure S7. Product profiles for the OXA-48 V120L variant with ertapenem, imipenem, panipenem, and biapenem.	S-9
Table S3. Chemical shift assignments for the imipenem-derived β-lactone products.	S-10
Figure S8 - S11. NMR spectra used for assignment of imipenem-derived β-lactone products.	S10- S14
Table S4. Chemical shift assignments for the panipenem-derived β-lactone products.	S-15
Figure S12 - S15. NMR spectra used assignment of panipenem-derived β-lactone products.	S16- S19
Figure S16. Product profiles for wild-type OXA-23, OXA-23 V128L, and OXA-23 V128I with meropenem.	S-20
Figure S17. Product profiles for the OXA-23 V120L variant with imipenem and panipenem.	S-21
Figure S18. The proposed, crystallographically observed conformation relevant to β - lactone formation in the active site of OXA-1	S-21
Figure S19. Leucine and isoleucine frequently occur at position 120 (using OXA-48 numbering) in class D SBLs.	S-22
References	S-23

A

OXA-48	Calculated mass (Da)	Observed mass (Da)
V120I	30844.9	30848.5
V120L	30844.9	30847.0
W105A	30715.8	30716.5
W105F	30791.9	30792.3
L158V	30816.9	30818.5
L158I	30830.9	30833.5

B

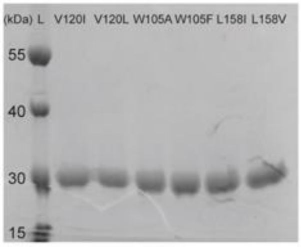

C

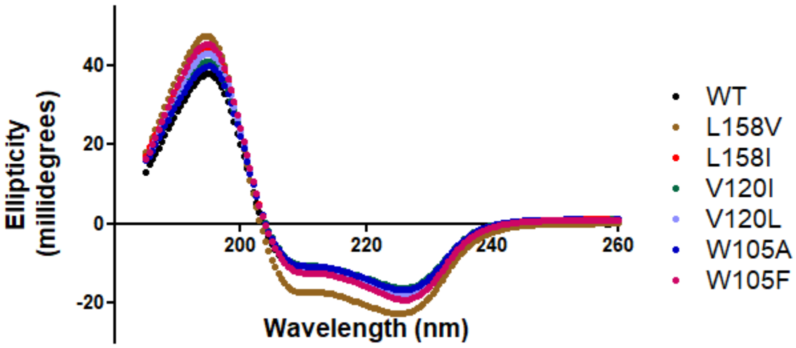

D

OXA-48	Melting temperature (°C)
wild-type	58.25 ± 0.07
V120I	56.64 ± 0.09
V120L	60.18 ± 0.1
L158I	58.79 ± 0.08
L158V	57.16 ± 0.06
W105A	51.91 ± 0.2
W105F	54.59 ± 0.1

Figure S1. Characterization of wild-type OXA-48 and variants. A. Mass spectrometric analyses of the N-terminally His_6-tagged OXA-48 variants. +177.8 Da and +257.9 Da adducts were observed, which likely correspond to α-N-6-phosphogluconoylation of the His-tag sequence, as previously reported (1). **B.** Coomassie-stained SDS-PAGE gel showing the purified recombinant OXA-48 variants. **C.** Circular dichroism (CD) spectra (n = 3 replicates, smooth = 10), indicating that the secondary structures of the OXA-48 variants resemble that of wild-type OXA-48. The CD spectrum for OXA-48 L158V differs slightly from the others, suggesting an increase in α-helicity. **D** Apparent melting temperatures of wild-type OXA-48 and variants, as determined by CD spectroscopy (n = 3 replicates). The melting curves are not reversible, because at higher temperature the proteins precipitated. The apparent melting temperatures of the variants are all similar, with the exception of OXA-48 W105A. Note, the CD spectrum for OXA-48 W105A is similar to that of wild-type OXA-48.

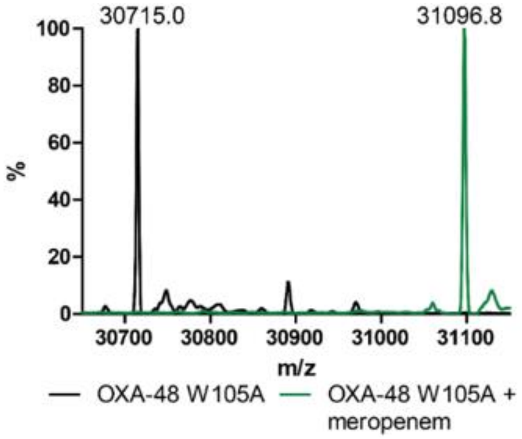

Figure S2. Mass spectra for OXA-48 W105A with meropenem. Deconvoluted positive ion electrospray ionization mass spectra of OXA-48 W105A alone (black) and with meropenem (green). These spectra imply that the W105A variant fully reacts with meropenem within 10 s under these conditions, forming a stable AEC. In combination with the kinetic studies described in the main text, these spectra suggest that the W105A substitution impacts on the deacylation step, with the acylation step being relatively unaffected.

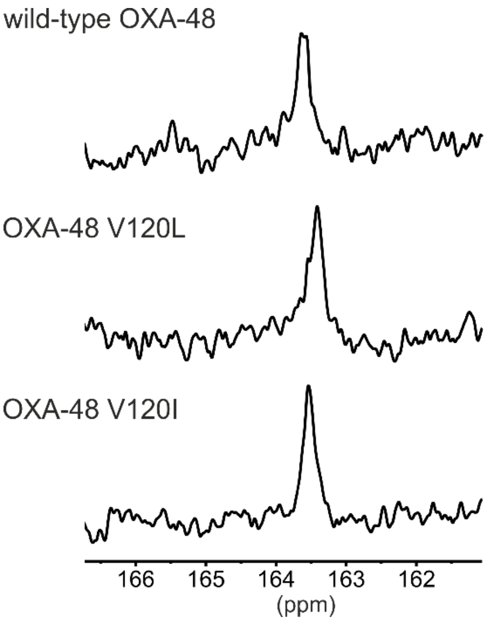

Figure S3. ^{13}C-NMR (150 MHz) spectra supporting lysine carbamylation of the OXA-48 V120I and V120L variants. The purified enzymes (700 µM) were treated with 10 mM $NaH^{13}CO_3$ in 50 mM sodium phosphate pH 7.5 using a previously reported procedure (2).

Table S1. Steady-state kinetics of wild-type OXA-48 and variants with nitrocefin in the presence and absence of bicarbonate. Assay conditions: nitrocefin (5 - 1500 µM) and enzyme (25-50 pM) in 50 mM sodium phosphate, pH 7.5, 0.01% Triton X-100, with and without 50 mM sodium bicarbonate. Errors are given as standard deviations, n = 3.

OXA-48	k_{cat} (s^{-1})	K_M (µM)	k_{cat}/K_M ($M^{-1}s^{-1}$) x 10^{-6}	Addition of bicarbonate
**wild-type**	593.2 ± 18.3	23.9 ± 3.5	24.8 ± 3.7	-
**V120L**	287.1 ± 11.7	184.1 ± 22.8	1.5 ± 0.2	
**V120I**	323.2 ± 11.1	40.1 ± 4.9	8.1 ± 1.0	
**wild-type**	663.2 ± 17.8	34.6 ± 3.7	19.1 ± 2.1	+
**V120L**	320.6 ± 79.6	275.9 ± 62.2	1.2 ± 0.4	
**V120I**	356.8 ± 15.5	330.8 ± 38.7	1.2 ± 0.1	

OXA-23	Calculated mass (Da)	Observed mass (Da)
V128I	31076.77	31079.5
V128L	31076.77	31078.8

Figure S4. Mass spectrometric analysis of the N-terminally His_6-tagged OXA-23 variants. +177.8 Da and +257.9 Da mass adducts were observed, which likely correspond to α-N-6-phosphogluconoylation of the His-tag (1).

Table S2. Summary of steady-state kinetic parameters for wild-type OXA-23 and variants with nitrocefin, meropenem, and imipenem. See the Experimental Procedures section for assay details; errors are given as standard deviations, n = 3.

OXA-23	substrate	k_{cat} (s^{-1})	K_M (μM)	k_{cat}/K_M ($M^{-1}s^{-1}$) x 10^{-6}
**wild-type**	nitrocefin	260.3 ± 5.9	93.2 ± 6.6	2.79 ± 0.2
	meropenem	0.063 ± 0.002	3.4 ± 0.4	0.019 ± 0.002
	imipenem	0.59 ± 0.03	5.9 ± 0.9	0.10 ± 0.02
**V128L**	nitrocefin	119.1 ± 10.4	437.4 ± 85.5	0.27 ± 0.06
	meropenem	0.14 ± 0.009	8.2 ± 2.3	0.017 ± 0.005
	imipenem	0.094 ± 0.005	5.0 ± 1.3	0.019 ± 0.005
**V128I**	nitrocefin	30.1 ± 2.4	499.8 ± 115.2	0.06 ± 0.02
	meropenem	0.066 ± 0.003	1.9 ± 0.5	0.035 ± 0.01
	imipenem	0.54 ± 0.007	9.4 ± 0.6	0.057 ± 0.003

A

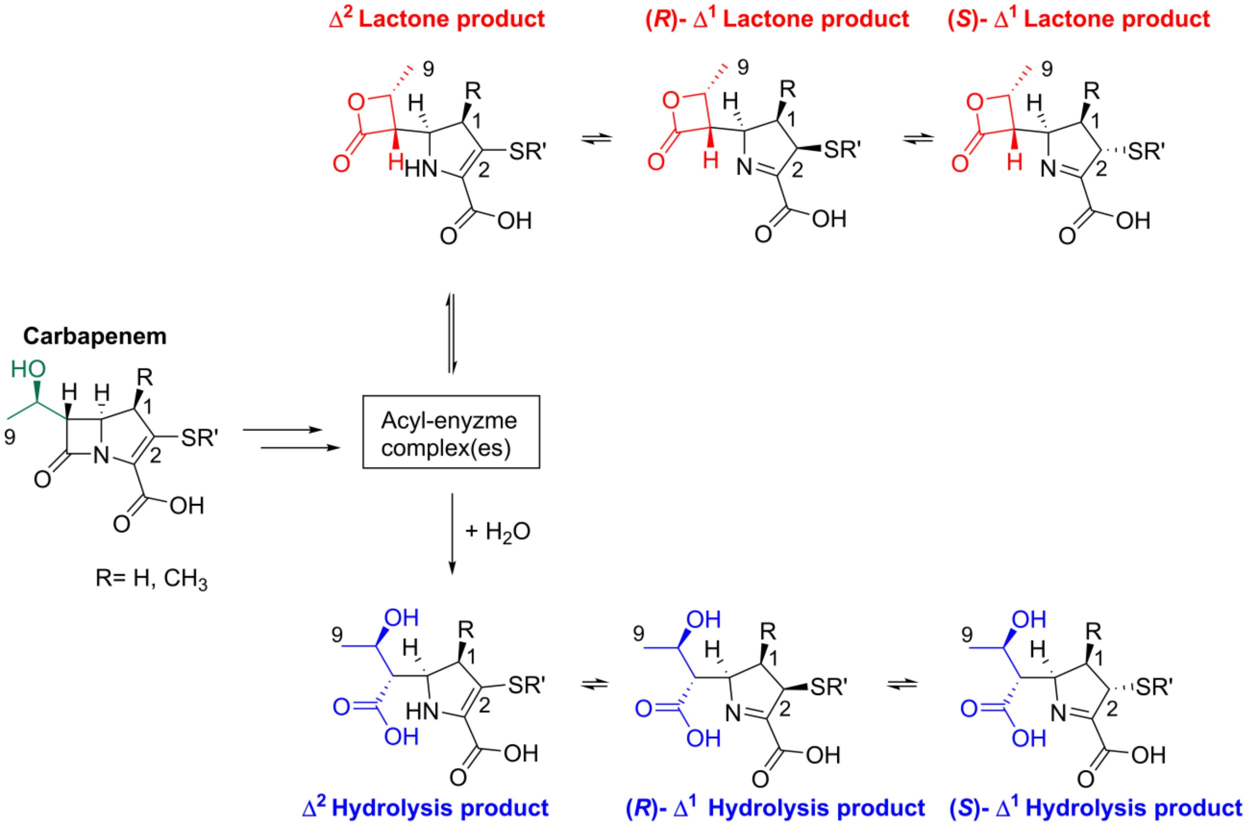

Δ2 Lactone product
(R)- Δ1 Lactone product
(S)- Δ1 Lactone product
Carbapenem
Acyl-enyzme complex(es)
+ H2O
R= H, CH3
Δ2 Hydrolysis product
(R)- Δ1 Hydrolysis product
(S)- Δ1 Hydrolysis product

B

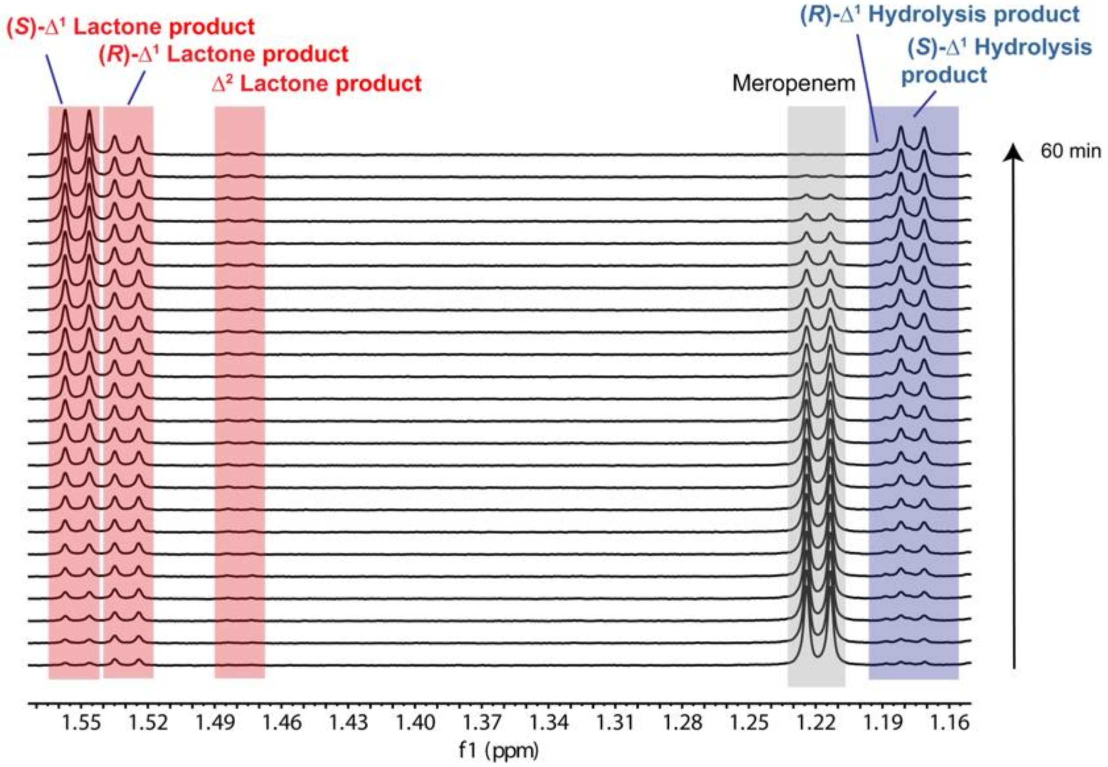

(S)-Δ1 Lactone product
(R)-Δ1 Lactone product
Δ2 Lactone product
Meropenem
(R)-Δ1 Hydrolysis product
(S)-Δ1 Hydrolysis product
60 min
1.55 1.52 1.49 1.46 1.43 1.40 1.37 1.34 1.31 1.28 1.25 1.22 1.19 1.16
f1 (ppm)

Figure S5. Carbapenem derived products formed by class D SBL catalysis. A. The pyrroline ring of the β-lactone and hydrolysis products occurs in three tautomeric / stereoisomeric forms. The Δ^2 (2-pyrroline) enamine form and the epimeric (*R*)-Δ^1 and (*S*)-Δ^1 (1-pyrroline) imine forms are in equilibrium; the Δ^2 enamine form is proposed to be the (major) nascent enzymatic product (3). **B.** Representative ^{1}H-NMR time course showing formation of the Δ^2 enamine (trace amount), the (*R*)-Δ^1 and (*S*)-Δ^1 imine β-lactones, and the (*R*)-Δ^1 and (*S*)-Δ^1 imine hydrolysis products derived from meropenem by wild-type OXA-48 over 60 min. The signals shown in these partial spectra correspond to the C-9 methyl group of the C-6 hydroxyethyl side chain. For the β-lactone products, it is proposed that the Δ^2 enamine form is the (major) nascent enzymatic product resulting from efficient turnover; this is proposed to rapidly (and non-enzymatically) tautomerize into the (*R*)-Δ^1 imine form, which then equilibrates over time to yield the more stable (*S*)-Δ^1 imine form. It was difficult to use NMR to analyze the *cis*-/ *trans*- stereochemistry of the imipenem or panipenem-derived β-lactones (due to signal overlap), thus we further analyzed previous NMR data for the two protons β-lactone ring in the β-lactone derived from the meropenem (4). The coupling constant between the two protons on the meropenem derived β-lactone is 6.8 Hz. We compared this with reported coupling constants for vittatalactone (*trans* -β-lactone; J = 4.0 Hz) and for obafluorin (*cis*- β-lactone; J = 6.8 Hz) (5, 6). These data support our assignment of the carbapenem-derived β-lactones as *cis*-β-lactones, i.e. indicate no or low levels (see Figure S8B) of epimerisation leading to *trans*- β-lactones. Note that hydrolyzed products, which do not react reversibly, predominate on prolonged incubation.

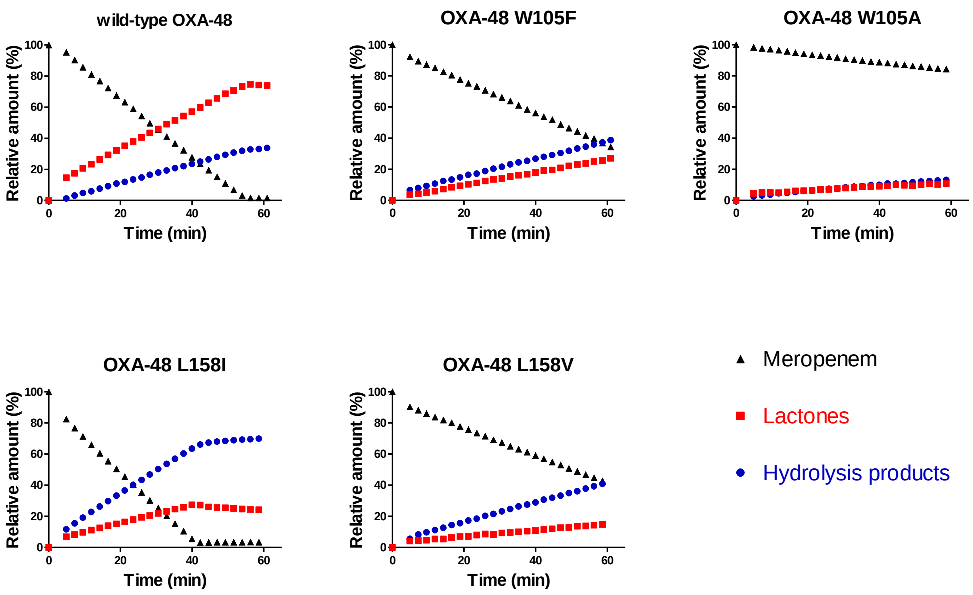

Figure S6. Product profiles for the OXA-48 W105A, W105F, L158I, and L158V variants with meropenem. ^{1}H NMR (600 MHz) time courses acquired over 60 min, showing the varying assigned products occurring for the OXA-48 variants (W105A, W105F, L158I, and L158V) with meropenem. Note the panel showing the data for wild-type OXA-48 of this figure is the same as in Figure 2A - it is included so comparison with the wild-type enzyme can be readily made. See the Experimental Procedures section for assay details.

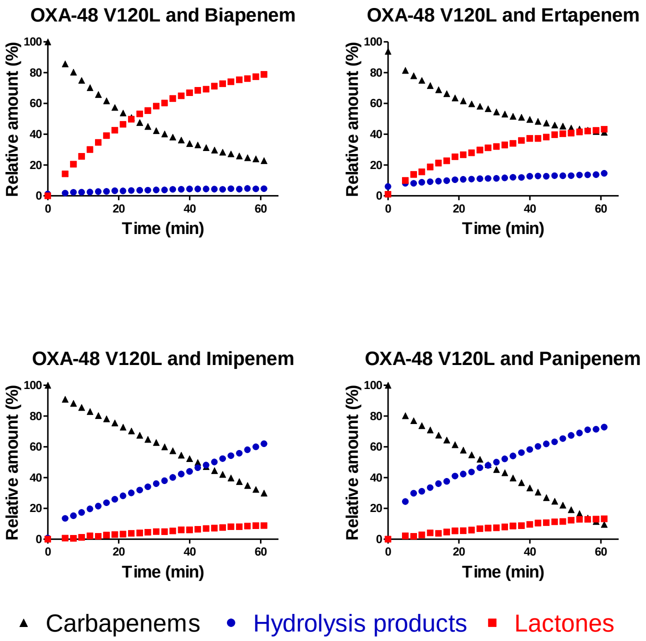

Figure S7. Product profiles for the OXA-48 V120L variant with ertapenem, imipenem, panipenem, and biapenem. ^{1}H NMR (600 MHz) time courses acquired over 60 min, showing the varying assigned products formed by the OXA-48 V120L variant with ertapenem, imipenem, panipenem and biapenem. See the Experimental Procedures section for assay details.

Table S3. Chemical shift assignments for the imipenem-derived β-lactone products.

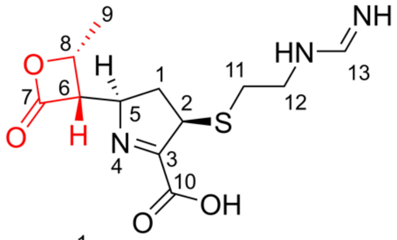

(*R*)-Δ[1]

Position	^{13}C (ppm)	^{1}H (ppm)
1	35.5	1.57, 2.82
2	49.6	4.18
3	174.8	
5	66.8	4.45
6	56.6	3.84
7	172.6	
8	73.4	4.94
9	15.1	1.55
11	28.4	2.86
12	40.7	3.50
13	154.7	7.74

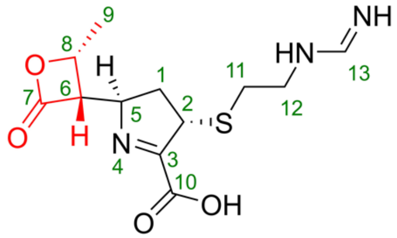

(*S*)-Δ[1]

Position	^{1}H (ppm)
1	2.24, 2.25
2	4.26
3	
5	4.59
6	3.82
7	
8	4.93
9	1.56
11	
12	
13	

Assignment of structures of the (*R*)-Δ[1] imine and (*S*)-Δ[1] imine of imipenem-derived β-lactone products. This assignment is supported by the distinctive chemical shifts of the protons at C-1 and C-2 (3), similar to what was previously observed for the imipenem hydrolysis product (3).

A

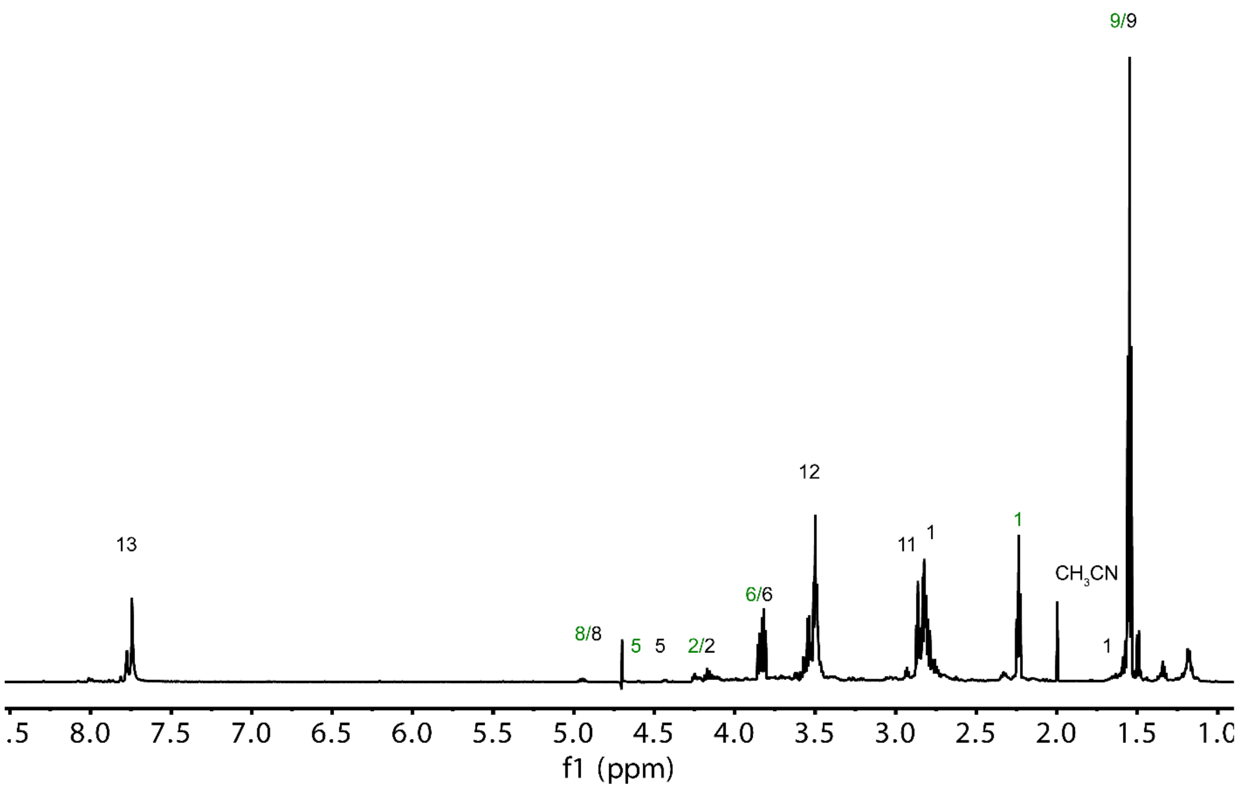

B

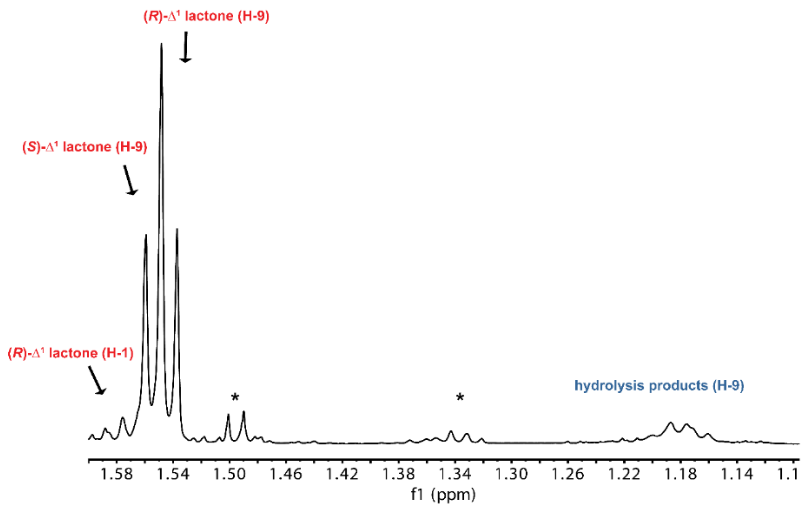

Figure S8. ^{1}H NMR (600 MHz) spectrum of the imipenem-derived β-lactone products. The β-lactones from OXA-48 V120L incubation were purified by HPLC as previously reported (4). In brief, a mixture of 10 mM imipenem or panipenem/25 µM OXA-48 V120L was incubated (room temperature, 4 h, in 50 mM sodium phosphate, pH 7.5). The tautomeric mixtures of β-lactones derived from imipenem and panipenem were optimally prepared using OXA-48 S70C (7), which makes the same β-lactone products derived from imipenem and panipenem as OXA-48 V120L

(details to be reported elsewhere). The enzyme was removed using an Amicon Ultra-0.5 mL centrifugal filter (3 kDa MW cut-off). The crude products were purified using a JASCO HPLC platform equipped JU-2086 plus preparative scale pumps; SunFire™ Prep C18 column (10 x 150 mm, 5 µm particle size; Waters). Mobile phases (A) 0.1 % aqueous trifluoroacetic acid (TFA), and (B) acetonitrile with 0.1 % aqueous TFA, at a flow-rate of 3 mL/min, monitoring at 220 nm, eluting with a gradient from 10 % B to 40 % B over 15 min. Collected fractions were frozen and lyophilized. NMR solvent:50 mM phosphate pH 7.5, 10% D_2O. **A.** The spectrum shows the presence of the (*R*)-Δ^1 imine form (black numbers) and the (*S*)-Δ^1 imine form (green numbers) of the imipenem-derived β-lactones and small amount of hydrolysis products. **B.** The selected spectrum of HPLC purified imipenem derived products showing the 1.60 -1.10 ppm region, i.e. the H-9 methyl group protons. The major isolated products are the (*R*)-Δ^1 imine β-lactone and the (*S*)-Δ^1 imine β-lactone. Due to the facile nature of the tautomerisation it was not possible to separate the (*R*)-Δ^1 imine form and the (*S*)-Δ^1 imine form. The minor products are likely the hydrolysis products. Trace amounts of peaks marked with * may correspond to stereoisomers of the N-formimidoyl side chain (8) or enamine product (though we cannot rule out the possibility of low levels of e.g., cis-β-lactone formation).

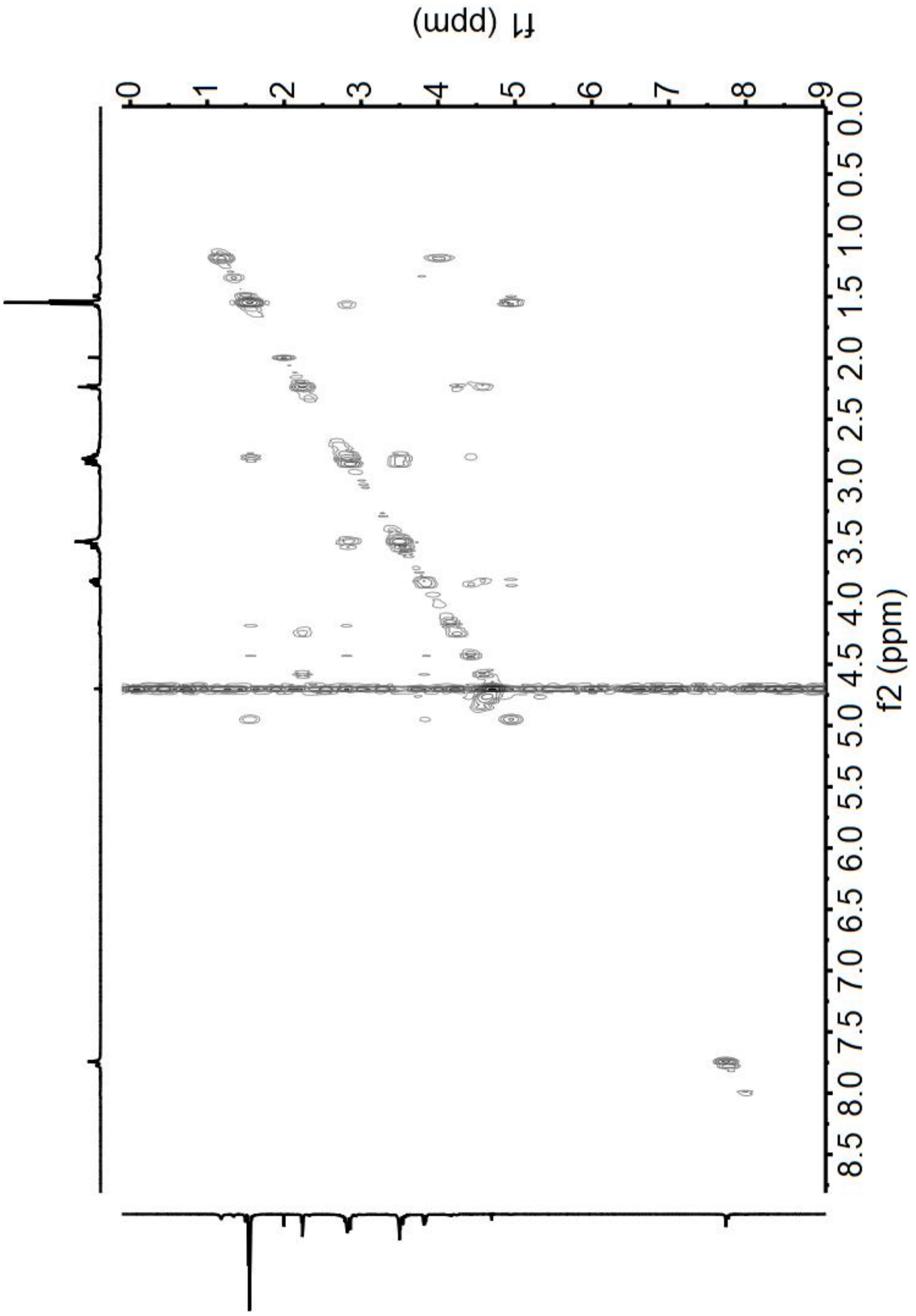

Figure S9. ^{1}H ^{1}H COSY spectrum (600 MHz) of the imipenem-derived β-lactone products. The β-lactones were purified by HPLC. Solvent: 50 mM phosphate pH 7.5, 10% D_2O.

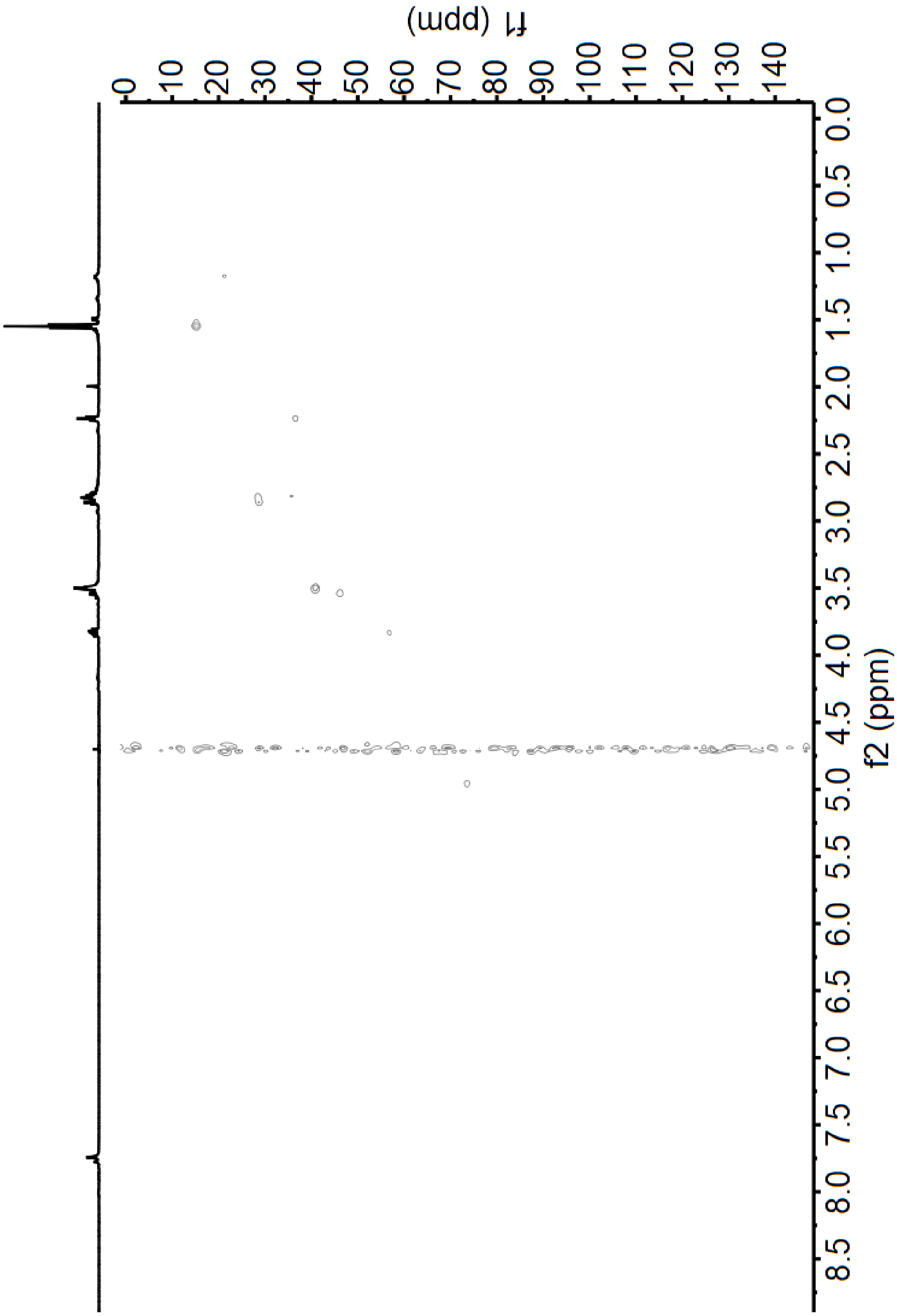

Figure S10. ^{1}H ^{13}C HSQC (600 MHz) spectrum of the imipenem-derived β-lactone products. The β-lactones were purified by HPLC. Solvent: 50 mM phosphate pH 7.5, 10% D_2O.

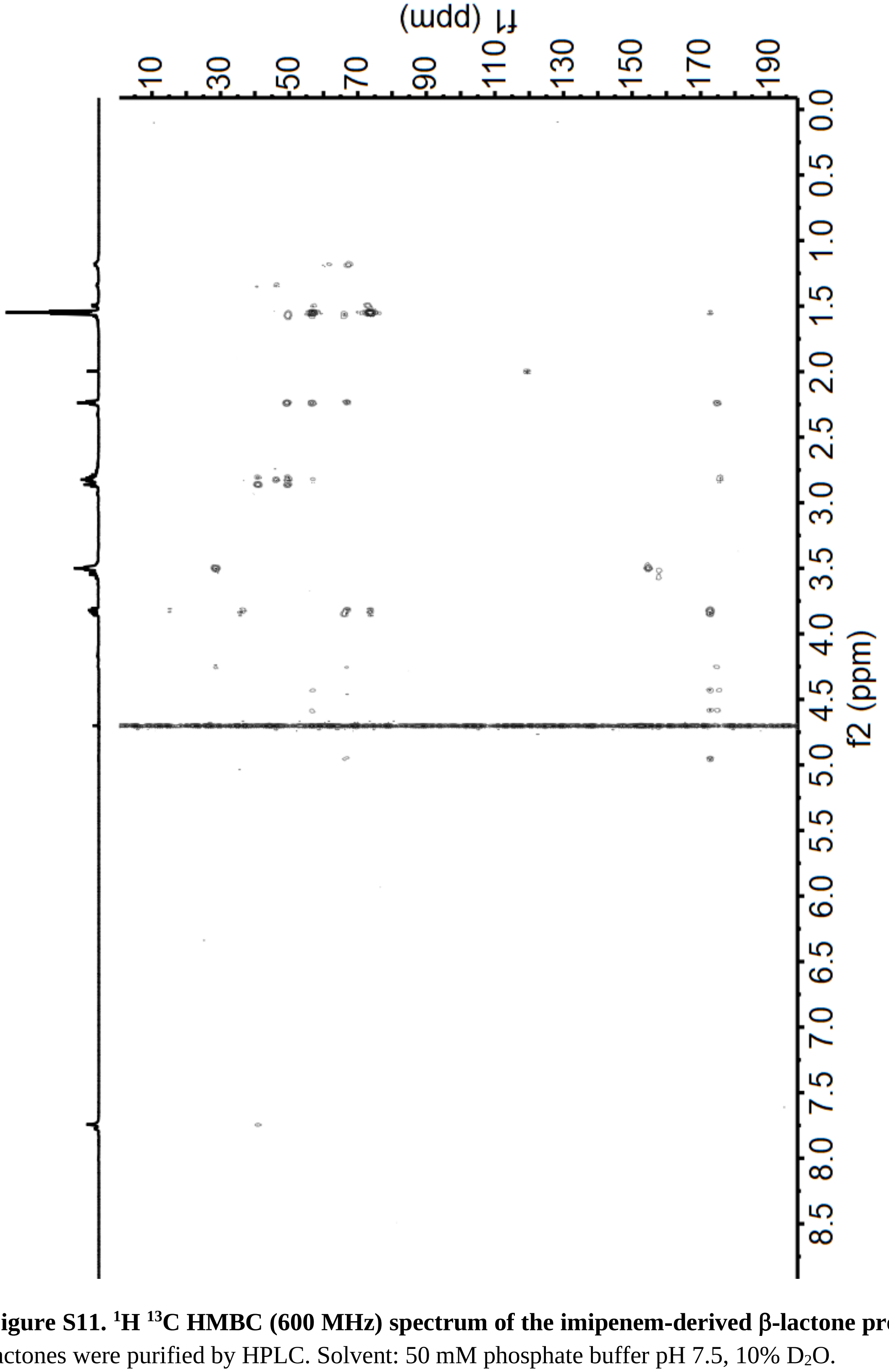

Figure S11. ¹H ¹³C HMBC (600 MHz) spectrum of the imipenem-derived β-lactone products. The β-lactones were purified by HPLC. Solvent: 50 mM phosphate buffer pH 7.5, 10% D_2O.

Table S4. Chemical shift assignments for the panipenem-derived β-lactone products.

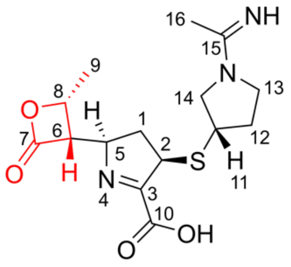

(*R*)-Δ^1

Position	^{13}C (ppm)	^1H (ppm)
1	36.4	1.57, 2.84
2	50.0	4.18
3	175.7	
5	66.7	4.42
6	56.5	3.82
7	172.3	
8	73.8	4.93
9	15.1	1.53
11	42.3	3.60
12	31.4	2.33, 1.96
13	154.7	3.42, 3.52
14	53.9	3.77, 3.29
15	163.0	
16	18.4	2.18

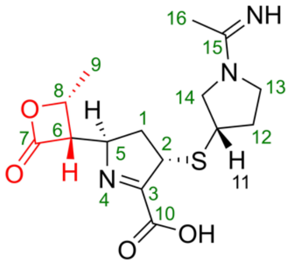

(*S*)-Δ^1

Position	^1H (ppm)
1	2.24, 2.25
2	4.26
3	
5	4.59
6	3.81
7	
8	4.93
9	1.54
11	
12	
13	
14	
16	

Chemical shift assignments of the (*R*)-Δ^1 imine and (*S*)-Δ^1 imine of panipenem-derived lactone products. These assignments are supported by the distinctive chemical shifts of the protons at C-1 and C-2, similar to what was previously observed for the imipenem hydrolysis product (3).

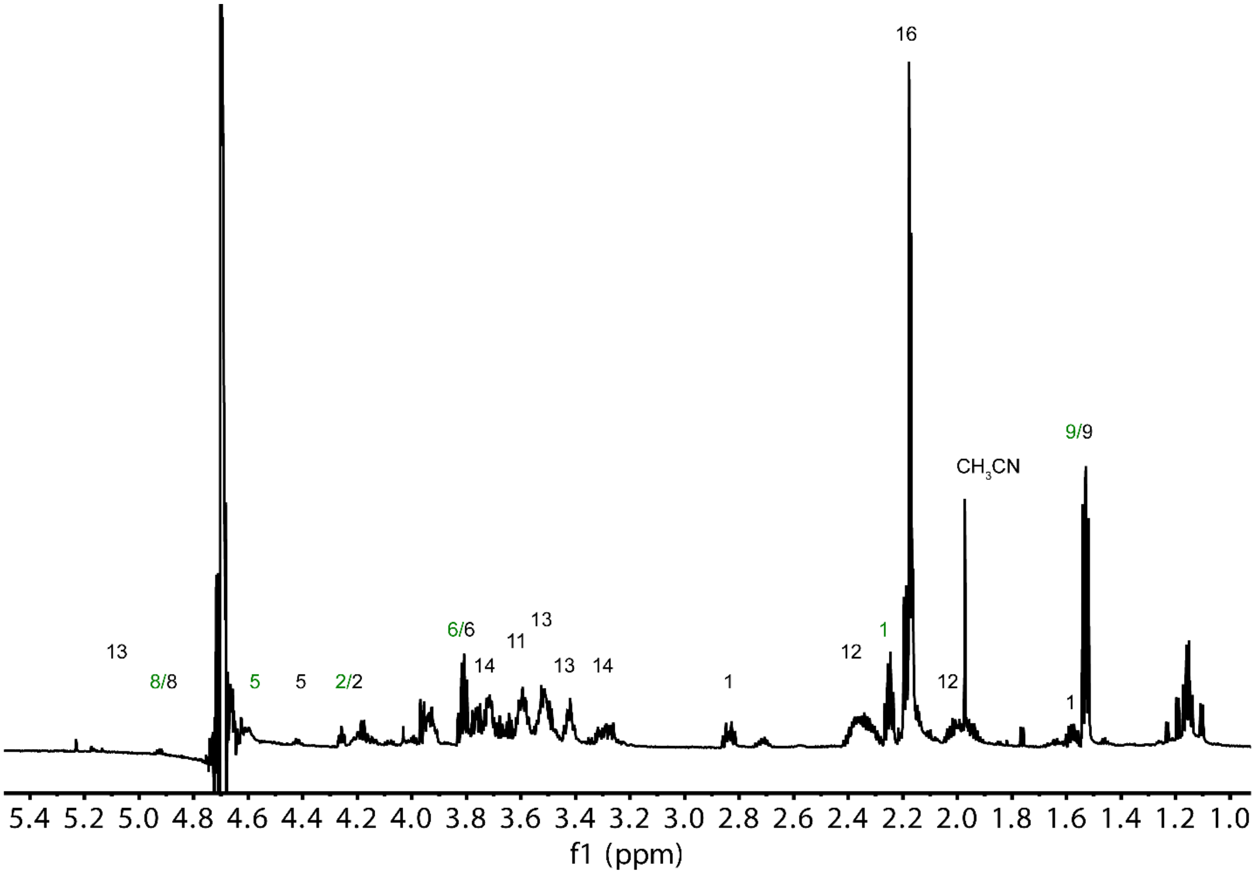

Figure S12. ^{1}H NMR (700 MHz) spectrum of the panipenem-derived β-lactone products. The β-lactones were purified by HPLC as previously reported (4)- see legend Figure S8. Solvent: 50 mM phosphate buffer pH 7.5, 10% D_2O. Note, the spectrum shows the presence of hydrolysis products, and the (*R*)-Δ^1 imine form and the (*S*)-Δ^1 imine form of the panipenem-derived lactones. The (*R*)-Δ^1 imine form and (*S*)-Δ^1 imine form are present in a similar amount, however, the precise ratio is difficult to analyze due to signal overlap with (*S*)-Δ^1 imine form and time-dependent formation.

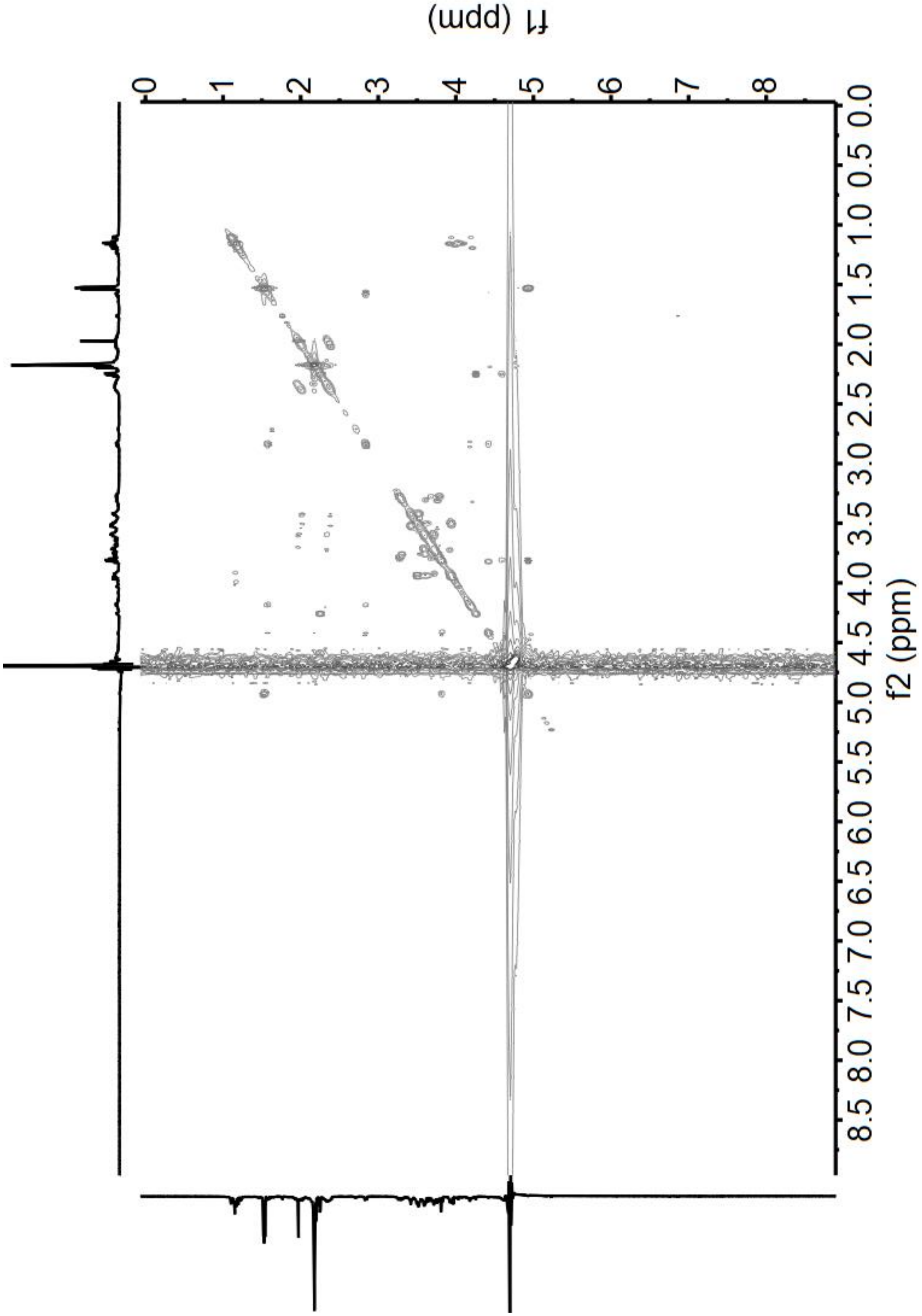

Figure S13. ^{1}H ^{1}H COSY (700 MHz) spectrum of the panipenem-derived β-lactone products. The β-lactones were purified by HPLC. Solvent: 50 mM phosphate buffer pH 7.5, 10% D_2O.

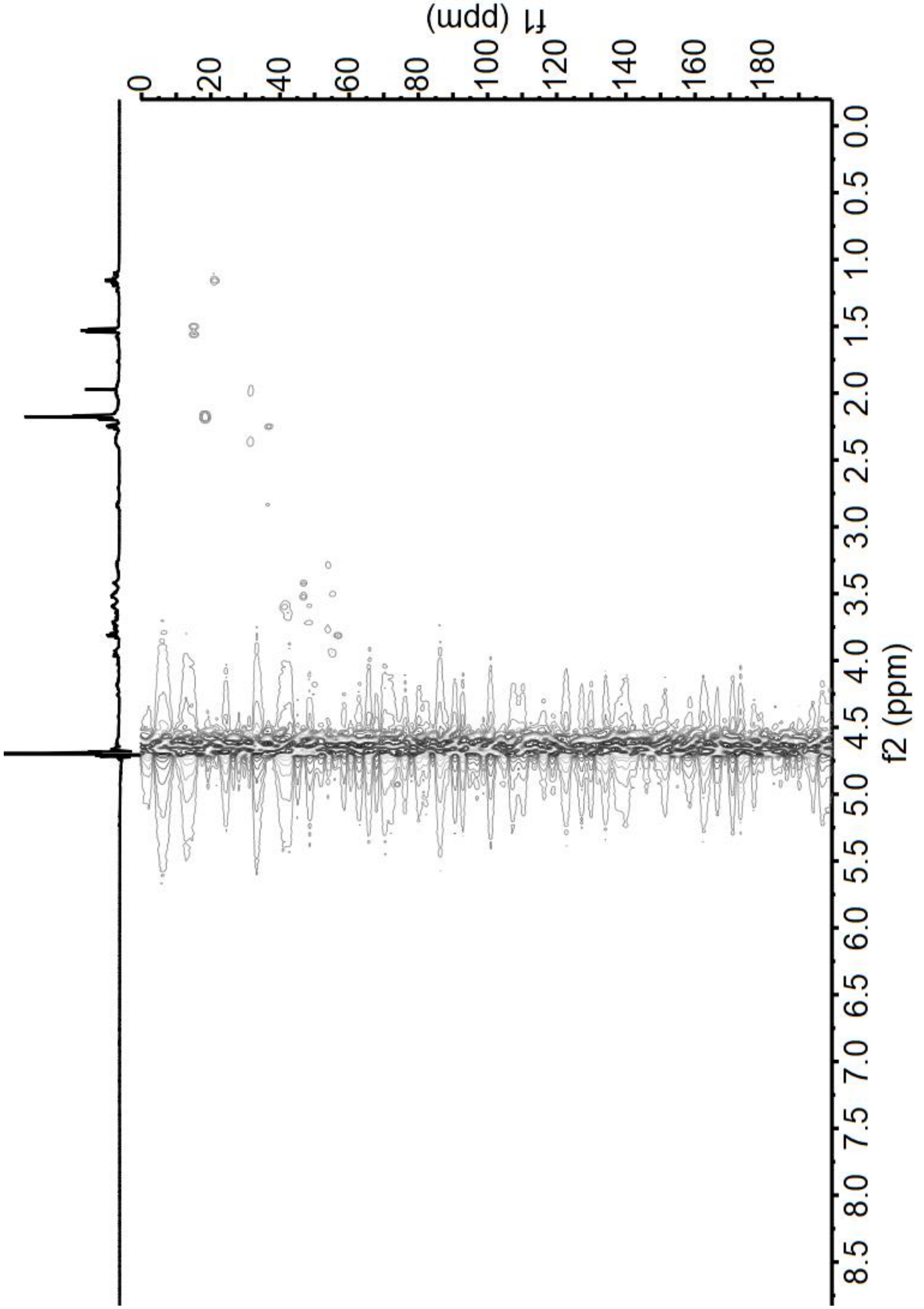

Figure S14. ¹H ¹³C HSQC (700 MHz) spectrum of the panipenem-derived β-lactone products. The β-lactones were purified by HPLC. Solvent: 50 mM phosphate buffer pH 7.5, 10% D_2O.

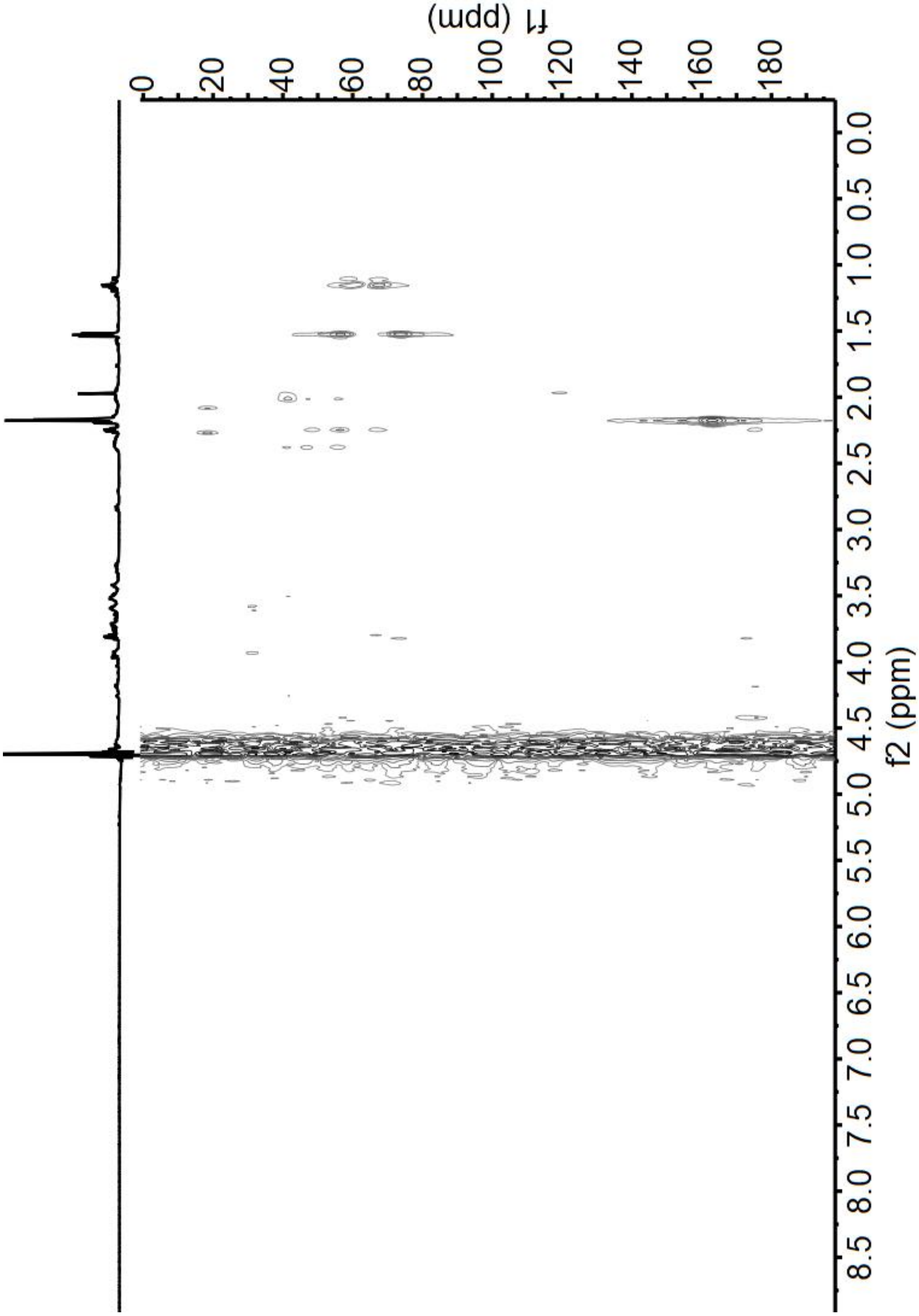

Figure S15. ^{1}H ^{13}C HMBC (700 MHz) spectrum of the panipenem-derived β-lactone products. The β-lactones were purified by HPLC. Solvent: 50 mM phosphate buffer pH 7.5, 10% D_2O.

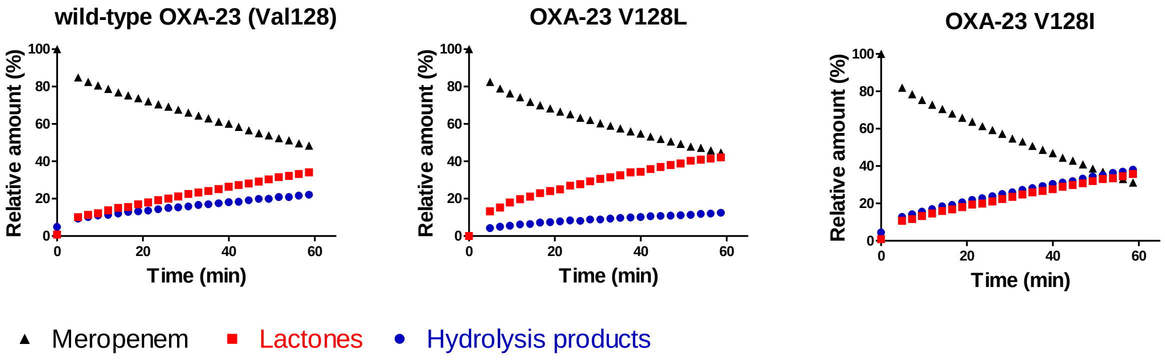

Figure S16. Product profiles for wild-type OXA-23, OXA-23 V128L, and OXA-23 V128I with meropenem. ^{1}H NMR (600 MHz) time courses acquired over 60 min, showing variations in the assigned products formed by wild-type OXA-23 and the OXA-23 V128L and V128I variants with meropenem. See the Experimental Procedures section for assay details.

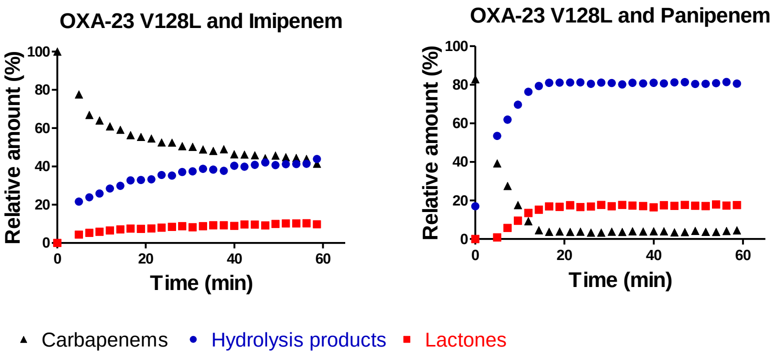

Figure S17. Product profiles for the OXA-23 V120L variant with imipenem and panipenem. ^{1}H NMR (600 MHz) time courses acquired over 60 min showing the products formed by the OXA-23 V128L variant with imipenem and panipenem. See the Experimental Procedures section for assay details.

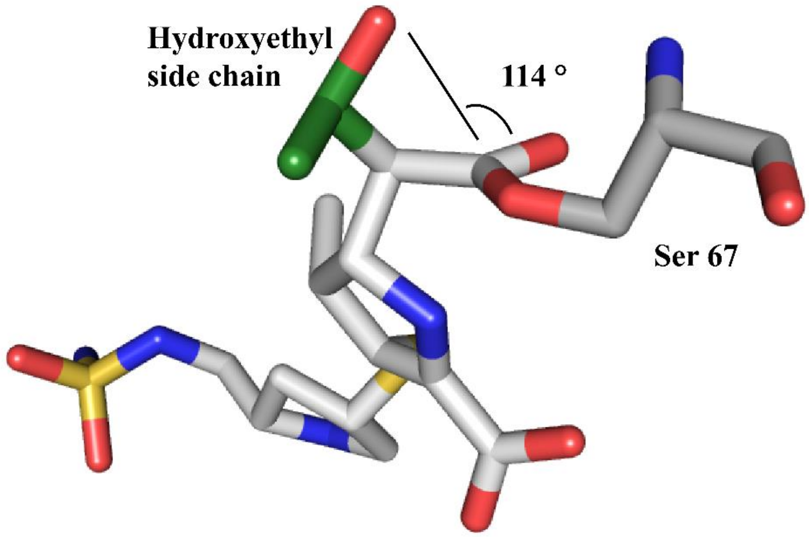

Figure S18. The proposed, crystallographically observed conformation relevant to β-lactone formation in the active site of OXA-1. A crystallographically derived view of the active site of OXA-1 after reaction with doripenem (PDB 3ISG) (9), indicating the ~114° angle of the C-6 hydroxyethyl hydroxyl group relative to the acyl-enzyme ester carbonyl group (described as conformation **I** in the main text, Figure 3). An angle of ~107° is considered optimal for nucleophilic attack leading to β-lactone formation (10).

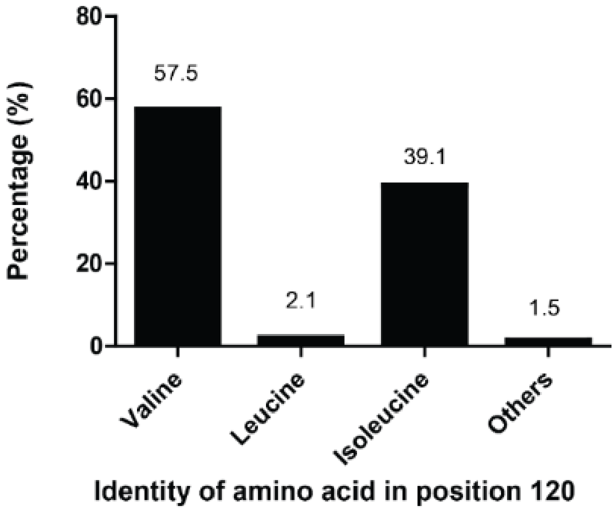

Figure S19. Leucine and isoleucine frequently occur at position 120 (using OXA-48 numbering) in class D SBLs. Analysis of the 932 class D SBLs listed in the Beta-Lactamase DataBase (BLDB) (11) shows that Val to Leu and Ile substitutions at position 120 are common (12). The Val-to-Ile substitution occurs in the OXA-51, OXA-213 and OXA-12 subfamilies. The Val-to-Leu substitution at position 120 is more widely distributed, occurring in eight different subfamilies.

References

1. Geoghegan, K. F., Dixon, H. B. F., Rosner, P. J., Hoth, L. R., Lanzetti, A. J., Borzilleri, K. A., Marr, E. S., Pezzullo, L. H., Martin, L. B., LeMotte, P. K., McColl, A. S., Kamath, A. V., and Stroh, J. G. (1999) Spontaneous α-N-6-Phosphogluconoylation of a "His Tag" inEscherichia coli:The Cause of Extra Mass of 258 or 178 Da in Fusion Proteins. *Analytical Biochemistry*. **267**, 169–184

2. Lohans, C. T., Wang, D. Y., Jorgensen, C., Cahill, S. T., Clifton, I. J., McDonough, M. A., Oswin, H. P., Spencer, J., Domene, C., Claridge, T. D. W., Brem, J., and Schofield, C. J. (2017) 13C-Carbamylation as a mechanistic probe for the inhibition of class D β-lactamases by avibactam and halide ions. *Org. Biomol. Chem.* **15**, 6024–6032

3. Lohans, C. T., Freeman, E. I., Groesen, E. van, Tooke, C. L., Hinchliffe, P., Spencer, J., Brem, J., and Schofield, C. J. (2019) Mechanistic Insights into β-Lactamase-Catalysed Carbapenem Degradation Through Product Characterisation. *Sci Rep*. **9**, 13608

4. Lohans, C. T., van Groesen, E., Kumar, K., Tooke, C. L., Spencer, J., Paton, R. S., Brem, J., and Schofield, C. J. (2018) A New Mechanism for β-Lactamases: Class D Enzymes Degrade 1β-Methyl Carbapenems through Lactone Formation. *Angew. Chem. Int. Ed.* **130**, 1296–1299

5. Morris, B. D., Smyth, R. R., Foster, S. P., Hoffmann, M. P., Roelofs, W. L., Franke, S., and Francke, W. (2005) Vittatalactone, a β-Lactone from the Striped Cucumber Beetle, Acalymma vittatum. *Journal of Natural Products*. **68**, 26–30

6. Tymiak, A. A., Culver, C. A., Malley, M. F., and Gougoutas, J. Z. (1985) Structure of obafluorin: an antibacterial .beta.-lactone from Pseudomonas fluorescens. *The Journal of Organic Chemistry*. **50**, 5491–5495

7. Lohans, C. T., Chan, H. T. H., Malla, T. R., Kumar, K., Kamps, J. J. A. G., McArdle, D. J. B., van Groesen, E., de Munnik, M., Tooke, C. L., Spencer, J., Paton, R. S., Brem, J., and Schofield, C. J. (2019) Non-Hydrolytic β-Lactam Antibiotic Fragmentation by l,d-Transpeptidases and Serine β-Lactamase Cysteine Variants. *Angew. Chem. Int. Ed*. **58**, 1990 –1994

8. Ratcliffe, R. W., Wildonger, K. J., Di Michele, L., Douglas, A. W., Hajdu, R., Goegelman, R. T., Springer, J. P., and Hirshfield, J. (1989) Studies on the structures of imipenem, dehydropeptidase I-hydrolyzed imipenem, and related analogs. *The Journal of Organic Chemistry*. **54**, 653–660

9. Schneider, K. D., Karpen, M. E., Bonomo, R. A., Leonard, D. A., and Powers, R. A. (2009) The 1.4 Å crystal structure of the class D β-lactamase OXA-1 complexed with doripenem. *Biochemistry*. **48**, 11840–11847

10. Bürgi, H. B., Dunitz, J. D., and Shefter, E. (1974) Chemical reaction paths. IV. Aspects of O...C = O interactions in crystals. *Acta Cryst.* **B30**, 1517–1527

11. Naas, T., Oueslati, S., Bonnin, R. A., Dabos, M. L., Zavala, A., Dortet, L., Retailleau, P., and Iorga, B. I. (2017) Beta-lactamase database (BLDB) – structure and function. *Journal of Enzyme Inhibition and Medicinal Chemistry*. **32**, 917–919

12. Evans, B. A., and Amyes, S. G. B. (2014) OXA β-Lactamases. *Clinical Microbiology Reviews*. **27**, 241–263
